# The roles of GpsB and DivIVA in *Staphylococcus aureus* growth and division

**DOI:** 10.3389/fmicb.2023.1241249

**Published:** 2023-08-30

**Authors:** Joshua A. F. Sutton, Mark Cooke, Mariana Tinajero-Trejo, Katarzyna Wacnik, Bartłomiej Salamaga, Callum Portman-Ross, Victoria A. Lund, Jamie K. Hobbs, Simon J. Foster

**Affiliations:** ^1^School of Biosciences, University of Sheffield, Sheffield, United Kingdom; ^2^The Florey Institute for Host-Pathogen Interactions, University of Sheffield, Sheffield, United Kingdom; ^3^Department of Physics and Astronomy, University of Sheffield, Sheffield, United Kingdom

**Keywords:** cell division, divisome, *Staphylococcus aureus*, GpsB, DivIVA, cell morphology

## Abstract

The spheroid bacterium *Staphylococcus aureus* is often used as a model of morphogenesis due to its apparently simple cell cycle. *S. aureus* has many cell division proteins that are conserved across bacteria alluding to common functions. However, despite intensive study, we still do not know the roles of many of these components. Here, we have examined the functions of the paralogues DivIVA and GpsB in the *S. aureus* cell cycle. Cells lacking *gpsB* display a more spherical phenotype than the wild-type cells, which is associated with a decrease in peripheral cell wall peptidoglycan synthesis. This correlates with increased localization of penicillin-binding proteins at the developing septum, notably PBPs 2 and 3. Our results highlight the role of GpsB as an apparent regulator of cell morphogenesis in *S*. *aureus*.

## Introduction

The cell envelope for most bacteria maintains cell shape and viability as well as forming an interface with the environment (Turner et al., 2014). In Gram-positive organisms, the envelope consists of a cell membrane containing lipoteichoic acids (LTAs) surrounded by a thick layer of peptidoglycan (PG) decorated with wall teichoic acids (WTAs) and surface proteins (Vollmer et al., 2008). The cell wall is dynamic, having to retain cellular integrity in the face of internal turgor while still permitting growth and division. In rod-shaped cells, the machinery required for vegetative growth is called the elongasome while that for cell division is the divisome (Cabeen and Jacobs-Wagner, 2005). Even the spheroid bacterium *Staphylococcus aureus* shows some elongation of the cell during growth but lacks an elongasome (Reichmann et al., 2019). Bacterial cell growth and division are highly organized and complex processes. PG structural dynamics are required for morphogenesis, with synthesis and hydrolysis being tightly controlled (Wheeler et al., 2015; Zhou et al., 2015). The final stages of PG synthesis are performed largely through the transglycosylase (TG) and transpeptidase (TP) activities of penicillin-binding proteins (PBPs) (Typas et al., 2011). *S. aureus* encodes four native PBPs, where PBP1 (monofunctional TP) and PBP2 (bifunctional TG and TP) are essential (Pinho and Errington, 2005; Wacnik et al., 2022). PBP1 has multiple functions during cell division, both enzymatically and as a scaffold (Wacnik et al., 2022). PBP3 is a non-essential TP (Pinho et al., 2000). The monofunctional TGs, FtsW and RodA, both of which are shape, elongation, division, and sporulation (SEDS) proteins, form cognate pairs with PBP1 (responsible for septum formation) and PBP3 (responsible for peripheral PG synthesis and cell shape maintenance), respectively (Reichmann et al., 2019). PBP4 has D,D-carboxypeptidase and TP activity, which results in the high level of PG crosslinking associated with *S. aureus* (Wyke et al., 1981; Atilano et al., 2010; Loskill et al., 2014). Methicillin-resistant *Staphylococcus aureus* (MRSA) strains possess an additional TP PBP2a, which has a low affinity for β-lactam antibiotics (Hartman and Tomasz, 1984; Pinho et al., 2001a).

Cell division is both spatially and temporally regulated to ensure the maintenance of cell shape and integrity. Staphylococcal cell division begins with the formation of the Z-ring, where multiple FtsZ monomers polymerise to form a scaffold to recruit divisome proteins that allow septation (Pinho et al., 2013; Szwedziak et al., 2014). Cell division must occur after DNA replication and subsequent chromosome segregation to ensure that the septa do not split the separating chromosomes. In part, this is achieved by proteins such as Noc (nucleoid occlusion factor), ParB, SMC, and CcrZ (Veiga et al., 2011; Chan et al., 2020; Gallay et al., 2021).

Bacterial cell division also requires the activity of many associated components, often of ill-defined function. DivIVA and GpsB are two divisome proteins that are conserved within Firmicutes, and the roles that they perform are well-reviewed (Halbedel and Lewis, 2019; Hammond et al., 2019). DivIVA is a coiled-coil protein, which binds to negatively curved membranes via its N-terminus, such as at the cell poles in rod-shaped organisms and where the septum crosses the cell (Lenarcic et al., 2009; Ramamurthi and Losick, 2009). The N-terminal domain is linked to the C-terminal domain via a short linker (Halbedel and Lewis, 2019), which facilitates oligomerisation into a tetramer (Muchová et al., 2002; Stahlberg et al., 2004; Rigden et al., 2008; Oliva et al., 2010). DivIVA localizes to the site of division in *Bacillus subtilis* forming two rings around the Z-ring. This prevents the Min system from interacting with FtsZ, allowing cell division to continue and stopping additional adjacent Z-rings from forming (Eswaramoorthy et al., 2011). DivIVA also interacts with the Spo0J/ParB system in *B. subtilis* and *Streptococcus pneumoniae* to facilitate chromosome segregation (Perry and Edwards, 2006; Fadda et al., 2007; Kloosterman et al., 2016). Little is known about the function of *S. aureus* DivIVA, with a null mutant having made no significant changes to cellular morphology or division (Pinho and Errington, 2004). DivIVA is stabilized through interacting with DnaK and plays a role in chromosome segregation, likely through an interaction with SMC (Bottomley et al., 2017).

GpsB is a homolog of DivIVA (Hammond et al., 2019). Both proteins have a similar overall structure, with a highly conserved N-terminal domain linked to a C-terminal domain (required for oligomerisation) via a short linker (Halbedel and Lewis, 2019). In *B. subtilis*, where it was first described, it was found to play a role in the switch between septal and peripheral peptidoglycan synthesis through interactions with PBP1 and MreC (Claessen et al., 2008; Tavares et al., 2008; Gamba et al., 2009). These observations suggest that GpsB acts as an adaptor protein to bring together different components of the divisome during the cell cycle (Cleverley et al., 2019). In *S. aureus*, it has been shown that GpsB interacts with and bundles FtsZ filaments, stabilizing the Z-ring and assisting with divisome recruitment (Eswara et al., 2018). GpsB also plays a role in linking cell division with wall teichoic acid display and synthesis (Hammond et al., 2022).

It has previously been reported that *gpsB* is essential (Eswara et al., 2018), and the function of *divIVA* remains relatively unknown in *S. aureus*. As *gpsB* is a homolog of *divIVA*, we aimed to find out whether these genes have a collective or distinct role in cell growth and division. In this study, we utilized super-resolution microscopy complemented with other molecular approaches to interrogate the function of *gpsB* and *divIVA* in *S. aureus*. We demonstrate that *gpsB* plays a role in cell shape determination.

## Materials and methods

### Bacterial growth conditions, plasmids, and oligonucleotides

The list of strains, plasmids, and oligonucleotides used in this study is listed in Supplementary Tables 1–3, respectively. All strains were cultured at 37°C with shaking at 200 rpm. For *S. aureus*, the mid-exponential phase was defined as an OD_600_ of 0.4–0.8. *E. coli* was cultured in Luria-Bertani (LB) broth or agar plus 100 μg/ml ampicillin. *S. aureus* strains were grown in tryptic soy broth (TSB) (Bacto) or agar (TSA), where required antibiotics were added at the following concentrations: 5 μg/ml erythromycin (Ery) 25 μg/ml lincomycin (Lin), 10 μg/ml chloramphenicol (Cm), 5 μg/ml tetracycline (Tet), 50 μg/ml kanamycin (Kan), and 100 μg/ml spectinomycin (Spec).

For growth curves, overnight *S. aureus* cultures were adjusted to OD_600_ 0.05 in TSB and incubated at 37°C with shaking at 200 rpm to grow for 8 h. Samples were taken every hour, and OD_600_ was measured. Direct cell counts were also performed by serial dilution in PBS and plating onto TSA. The number of colony-forming units (CFU) was directly counted after incubation. Growth curves were performed in triplicate. *E. coli* transformations and DNA manipulations were performed according to the previously described methodology (Sambrook and Russell, 2001).

### Construction of *S. aureus* mutants

Unless otherwise stated, all vectors were constructed in *E. coli* NEB5α (New England Biolabs, Ipswich, MA, United States) following previously described methods (Gibson et al., 2009; Lund et al., 2018) before passage through *S. aureus* RN4220 for DNA methylation (Novick and Morse, 1967). Finally, constructs were transduced into *S. aureus* SH1000 using phage Φ11. Transductions and transformations were confirmed by PCR. The genomic DNA of SH1000 was used as a template for *S. aureus* gene amplification. Genomic DNA was isolated by incubating *S*. *aureus* cells in 2.5 μg/ml lysostaphin prior to extraction using a Qiagen DNeasy Blood & Tissue Kit (Cat no. 69506) in accordance with the manufacturer's instructions. *S*. *aureus* transposon mutants were obtained from the NARSA library (Bae et al., 2008; Fey et al., 2013). Transposons were transduced from the library to the recipient strain and confirmed by PCR.

#### SH1000 *gpsB::kan*

To delete native *gpsB*, fragments encompassing 1,000 bp upstream and downstream of *gpsB* were amplified using oligos piMAY*_gpsB_up_F/R* and piMAY*_gpsB_down_F/R*. A kanamycin resistance cassette was amplified from pGL433 (Wheeler et al., 2015) using oligos *pGL433_kan_F/R* and included in between the upstream and downstream fragments to allow the selection of deletion mutants. The products were ligated into piMAY cut with KpnI and NotI, and fragments were combined using Gibson assembly creating piMAY *gpsB-ko*. The plasmid was electroporated into RN4220 at 30°C. The plasmid was integrated through a single-crossover event at 37°C, and the chromosomal DNA fragment containing the deletion cassette was transduced into SH1000 to produce SJF4925. Colonies were selected based on kanamycin resistance and tetracycline sensitivity.

#### SH1000 Δ*divIVA—pMAD*

To construct pMAD Δ*divIVA*, 1000 bp upstream and downstream of *divIVA* was amplified using oligo pairs pMAD_*divIVA_1/2* and *pMAD*_*divIVA_3/4*. pMAD was cut with BglII and EcoRI. The fragments were combined using Gibson assembly producing pMAD-Δ*divIVA*. This construct was transformed into RN4220, and a single-crossover event occurred. The integrated pMAD-Δ*divIVA* was transduced into SH1000 where pMAD was excised by double crossover as previously described (Arnaud et al., 2004), which produced strain SJF4814.

#### SH1000 *gpsB::gpsB-mCherry*

A C-terminal fusion of GpsB with mCherry was designed in pOB (Horsburgh et al., 2002b) and synthesized by GENEWIZ UK Ltd, Hertfordshire, United Kingdom. The synthesized plasmid was directly transformed into RN4220 (where it integrated into the chromosome through a single-crossover event) and transduced into SH1000 (selecting strains that were Kan^R^ but sensitive to Ery) to produce strain SJF5643.

#### SH1000 Δ*divIVA geh::divIVA-GFP*

A C-terminal fusion of DivIVA with GFP was created within pKASBAR *tet* (Bottomley et al., 2014). The insert region was synthesized by GENEWIZ UK Ltd and amplified using oligos pKB*-divIVA-F/-R*. The amplified fragment was then ligated into pKASBAR *tet* cut with BamHI and EcoRI using Gibson Assembly (creating pKASBAR*-divIVA-gfp*). The plasmid was transduced into RN4220 with integration at the *geh* locus being confirmed by disruption of lipase production on Baird-Parker medium and PCR. The chromosomal fragment was then transduced into SJF4814 (SH1000 Δ*divIVA*) to produce strain SJF5299.

#### SH1000 *gpsB::gpsB-mCherry kanR* Δ*divIVA geh::divIVA-megfp*

The chromosomal *gpsB::gpsB-mCherry* was transduced into SJF5299 and confirmed by PCR to produce SJF5669.

#### SH1000 Δ*divIVA geh::divIVA*

The *divIVA* locus including the native promoter (178 bp upstream) was cloned into pKASBAR *tet*. DNA fragments was made using oligos pKASBAR*_divIVA_F/R*. The resulting fragment was ligated into pKASBAR *tet* cut with BamHI and EcoRI using Gibson Assembly creating pKASBAR-*divIVA*. The construct was electroporated into RN4220, with integration at the *geh* locus being confirmed by disruption of lipase production on Baird-Parker medium and PCR. The chromosomal fragment containing integrated pKASBAR-*divIVA* was transduced into SJF4814 to produce SJF4899.

#### SH1000 *gpsB::kan geh::gpsB*

The *gpsB* locus including the native promoter was cloned into pKASBAR *tet*. DNA fragments were made using oligos pKASBAR*_gpsB_F/R*. The resulting fragment was ligated into pKASBAR *tet* cut with BamHI and EcoRI using Gibson assembly creating pKASBAR-*gpsB*. The construct was electroporated into RN4220, with integration at the *geh* locus being confirmed by disruption of lipase production on Baird-Parker medium and PCR. The chromosomal fragment containing integrated pKASBAR-*gpsB* was transduced into SJF4925 to produce SJF4956.

#### SH1000 *pbp3::spec* pLOW-*Ppcn-gfp-pbp3*

An N-terminal fusion of PBP3 with GFP was synthesized and cloned into pLOW under the control of a *Ppcn* promoter by GENEWIZ UK Ltd. This plasmid was electroporated into RN4220 and then transduced into SH1000 *pbp3::spec* and SH1000 *pbp3::spec gpsB::kan* to produce SJF5950 and SJF5951, respectively.

### Transmission electron microscopy

TEM was performed as mentioned in a study by Sutton et al. (2021). In brief, samples were fixed overnight in 2.5% (w/v) glutaraldehyde at 4°C. Samples were washed in PBS, and secondary fixation was performed with 2% (w/v) osmium tetroxide for 2 h. After washing, samples were dehydrated in incrementally increasing concentrations of ethanol and then incubated in propylene oxide. Samples were infiltrated overnight in a 50% (v/v) propylene oxide to 50% (v/v) Epon resin mixture overnight, which was then replaced with pure Epon resin for 4 h, which was then replaced with fresh resin for another 4 h. Polymerisation was then performed in fresh resin at 60°C for 48–72 h. Approximately 80 nm thin sections were taken and stained with 3% (w/v) aqueous uranyl acetate followed by Reynold's lead citrate. Sections were imaged using a FEI Tecnai T12 Spirit transmission electron microscope operating at 80 kV. Images were recorded using a Gatan Orius SC1000B bottom-mounted CCD camera. TEM images were analyzed using Fiji software (Schindelin et al., 2012). Cell wall thickness was measured as previously described by Sutton et al. (2021).

### Labeling of strains for fluorescence microscopy

*S. aureus* strains were grown overnight in TSB (with appropriate antibiotics), which were used to inoculate fresh TSB to an OD_600_ of 0.05. Cells were then grown to the mid-exponential phase (OD_600_ of ~0.5) before being labeled. Samples were protected from light throughout the staining process until imaged. Then, 500 μM of 7-hydroxycoumarin-3-carboxylic acid-amino-D-alanine (HADA) or 1mM of azido-Dalanyl-D-alanine (ADA-DA) (Kuru et al., 2012b; Monteiro et al., 2015; Lund et al., 2018) was added to cells for 5 or 30 min and incubated at 37°C with shaking to label newly synthesized peptidoglycan. Cells were then washed twice in PBS at 4°C. The azide group of ADA-DA was labeled (post-fixation) with 5 μg ml^−1^ Alexa Fluor 488 Alkyne using the Click-iT™ Cell Reaction Buffer Kit (Invitrogen) according to the manufacturer's instructions. To label and visualize the entire cell wall, cells were resuspended in PBS and incubated at 4°C for 5 min with 8 μg ml^−1^ Alexa Fluor 555 NHS ester (Invitrogen, Waltham, MA, United States). Cells were then washed in PBS. After labeling, cells were incubated with 2% (w/v) paraformaldehyde (PFA) for 30 min at room temperature and then washed twice in water. After fixation, cells were resuspended in water containing 2 μg/ml DAPI (Sigma) for 5 min at room temperature on a rotary shaker to visualize DNA where appropriate. Samples were then washed twice in water before mounting.

### Widefield epifluorescence microscopy

Cells were mounted onto poly-L-Lysine coated slides (Sigma) using SlowFade^TM^ Gold antifade reagent (Thermo Fisher Scientific, Waltham, MA, United States) and then imaged using a Nikon Ti inverted microscope fitted with a Lumencor Spectra X light engine. Images were obtained with a 100x PlanApo (1.4 NA) oil objective 1.518 RI oil, and an Andora Zyla sCMOS camera was used for detection.

### OMX structured illumination microscopy (SIM)

SIM was performed as previously described by Lund et al. (2018). In brief, coverslips (High-precision, 1.5H, 22 ± 22 mm, 170 ± 5 mm, Marienfeld) were sonicated in 1 M KOH for 15 min before being washed and then incubated in poly-L-Lysine solution for 30 min. Coverslips were then washed and dried before fixed cells (suspended in water) were dried onto coverslips and mounted with SlowFade^TM^ Gold antifade reagent (Thermo Fisher Scientific).

SIM was performed using a v4 DeltaVision OMX 3D-SIM system fitted with a Blaze module (Applied Precision, GE Healthcare, Issaquah, USA) with lasers used to illuminate samples. For each Z-slice (0.125 nm), images were taken in five phase shifts and three angles. To reconstruct images, the software Softworx (GE Healthcare, Issaquah, USA) was used with optimisation for a 1.516 immersion oil. The same software was used for deconvolution and image alignment.

### Microscopy analysis

All measurements from microscopy images were made using Fiji (Schindelin et al., 2012). Unless otherwise stated, micrographs presented are maximum intensity projections of Z-stacks.

#### Cell volume analysis

Cell volume analysis from widefield microscopy and SIM was analyzed as previously described (Zhou et al., 2015). Long- and short-axis measurements were taken for each cell, and the volume was calculated using the equation for the volume of a prolate spheroid:


$$\begin{array}{l} V=\frac{4}{3}\pi ab^{2} \end{array}$$

#### Cell elongation short/long cell axis ratio

The axis ratio was adapted from a previously published methodology (Reichmann et al., 2019). To calculate the short/long-axis cell ratio as an indicator of cell shape, the short-axis measurement of each cell (as taken for volume) was divided by the long axis to give a ratio where cells are perfectly circular at 1.0 and more elongated as the value decreases.

#### Fluorescence ratio septal/peripheral

The fluorescence ratio (FR) was calculated as previously published (Tinajero-Trejo et al., 2022). FR was calculated using fluorescence at the septum of cells with an incomplete septum with fluorescence measured between the cell periphery and the annulus. This was divided by the mean fluorescence at the lateral cell walls.

### Statistical analysis

Statistical analysis was performed using Prism version 9.31 (GraphPad, Boston, MA).

## Results

### GpsB and DivIVA localize at the septum

**Figure 1:**
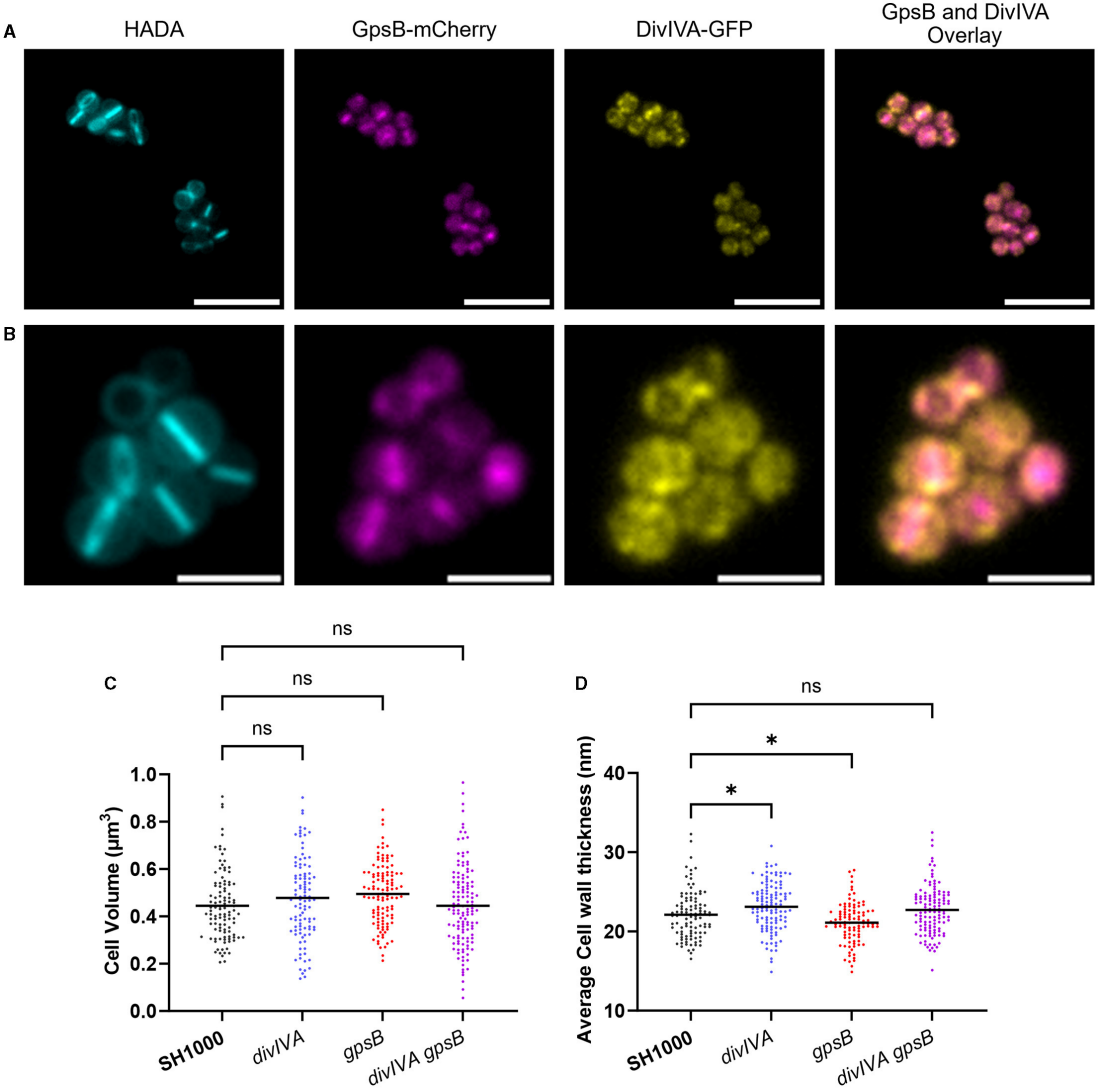
Localization and role of DivIVA and GpsB. **(A, B)** Representative fluorescence microscopy images of SH1000 cells labeled with HADA for 30 min and expressing GpsB-mCherry and DivIVA-GFP. An overlay of the localisations of GpsB-mCherry and DivIVA-GFP is also shown [a scale bar for **(A)** represents 5 μm, and a scale bar for **(B)** represents 2 μm)]. **(C)** Cell volume analysis from SIM micrographs of SH1000 (black circles, *n* = 107 cells), *divIVA* (blue circles, *n* = 104 cells), *gpsB* (red circles, *n* = 114 cells), and *divIVA gpsB* (purple circles, *n* = 130 cells). **(D)** Average cell wall thicknesses of SH1000 (black circles, *n* = 103 cells), *divIVA* (blue circles, *n* = 120 cells, ^*^p = 0.0283), *gpsB* (red circles, *n* = 101 cells, ^*^p = 0.0401), and *divIVA gpsB* (purple circles, *n* = 119 cells). The results for **(B, C)** were analyzed using a one-way ANOVA with multiple comparisons (ns *p* > 0.05).

As DivIVA and GpsB are paralogues, they may be functionally related and therefore share a localization. A previous study has independently shown that DivIVA (Pinho and Errington, 2004) and GpsB (Eswara et al., 2018) localize to the *S. aureus* septum. To assess the co-localization of GpsB and DivIVA fusion, constructs were produced using pOB (GpsB-mCherry) and pKASBAR (DivIVA-GFP) and co-expressed in the same strain (SH1000 *gpsB::gpsB-mCherry kanR* Δ*divIVA geh::divIVA-gfp*). Both GpsB-mCherry and DivIVA-GFP localize at the septum when compared to a HADA label, which allows visualization of new cell wall peptidoglycan synthesis by fluorescence microscopy (Kuru et al., 2012a) (Figures 1A, B). However, DivIVA-GFP has a ‘dotty' pattern of localization, which has previously been reported (Pinho and Errington, 2004), whereas GpsB-mCherry is seen to be forming smooth rings, suggesting that these proteins are not co-localizing. When viewing through the Z-stacks, it is clear that both GpsB-mCherry and DivIVA-GFP are forming independent patterns at the developing septum (Supplementary Video 1).

### *gpsB* and *divIVA* mutations do not impact cell volume

Previous reports have suggested that GpsB is an essential protein in *S. aureus* (Santiago et al., 2015; Eswara et al., 2018); however, in this study, we were able to construct a marked deletion of the *gpsB* gene in SH1000 using piMay, and thus, it is non-essential in this background. A transposon inactivation *gpsB* mutant can also be found within the NARSA transposon library (Fey et al., 2013). A markerless *divIVA* deletion mutant was also constructed using pMAD, as well as a double mutant (SH1000 *gpsB::kan* Δ*divIVA*). No differences in growth were found between any of the mutants or when compared to the wild type (Supplementary Figure 1). Structured illumination microscopy (SIM) was used to analyse the cell volume of the mutants (Figure 1C and Supplementary Figure 2). No significant differences were observed in the volumes of the SH1000 Δ*divIVA*, SH1000 *gpsB::kan*, or SH1000 Δ*divIVA gpsB::kan* strains compared to the wild-type SH1000. Next, TEM was used to interrogate the cell wall structure of these mutants (Figure 1D and Supplementary Figure 1). *divIVA* was found to have a slightly thicker peripheral cell wall than wild-type cells (*p* = 0.0283), while *gpsB* had a slightly thinner cell wall (*p* = 0.0401). No difference in cell wall thickness was observed in *divIVA gpsB* compared to wild-type SH1000 (Figure 1D).

### Combinatorial mutagenesis reveals a link for *gpsB* and *divIVA* with teichoic acid synthesis and display

The roles of *gpsB* and *divIVA* in other species and the results of bacterial two-hybrid screens previously described in the literature (Steele et al., 2011; Bottomley et al., 2017) allowed us to determine a potential role for DivIVA and GpsB in various aspects of cell growth and division. As single and double mutants of *gpsB* and *divIVA* did not show any clear phenotype, further mutations, from the NARSA transposon library (Fey et al., 2013) (unless otherwise stated), were added in combination with *divIVA gpsB* to provide information about DivIVA and GpsB.

**Figure 2:**
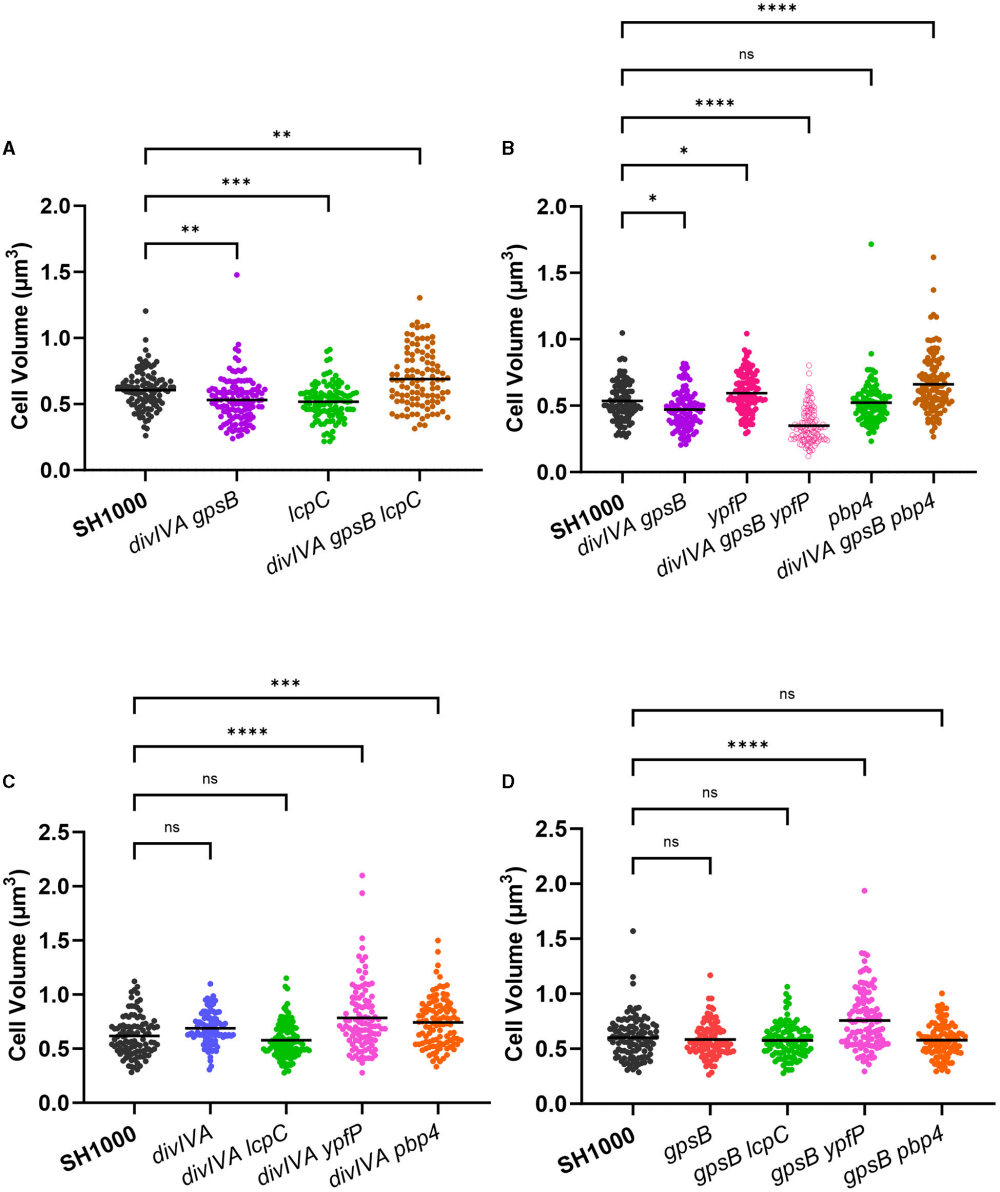
Functional interaction between DivIVA and GpsB with other components. **(A)** Cell volume analysis from SIM micrographs of SH1000 (black circles, *n* = 104 cells), *divIVA gpsB* (purple circles, *n* = 124 cells, ^**^p = 0.0028), *lcpC* (green circles, *n* = 107 cells, ^***^p = 0.0004), and *divIVA gpsB lcpC* (brown circles, *n* = 104 cells, ^**^p = 0.0014). **(B)** Cell volume analysis from SIM micrographs of SH1000 (black circles, *n* = 105 cells), *divIVA gpsB* (purple circles, *n* = 107 cells, ^*^p = 0.0192), *ypfP* (pink circles, *n* = 113 cells, ^*^p = 00348), *divIVA gpsB ypfP* (open pink circles, *n* = 117 cells, ^****^p < 0.0001), *pbp4* (green circles, *n* = 102 cells, ns *p* = 0.9711), and *divIVA gpsB pbp4* (brown circles, *n* = 125 cells, ^****^p < 0.0001). **(C)** Cell volume analysis from SIM micrographs of SH1000 (black circles, *n* = 104 cells), *divIVA* (blue circles, *n* = 102 cells, ns *p* = 0.0673), *divIVA lcpC* (green circles, *n* = 119 cells, ns *p* = 0.4148), *divIVA ypfP* (pink circles, *n* = 105 cells, ^****^p < 0.0001), and *divIVA pbp4* (orange circles, *n* = 101 cells, ^***^p = 0.0002). **(D)** Cell volume analysis from SIM micrographs of SH1000 (black circles, *n* = 108 cells), *gpsB* (red circles, *n* = 100 cells, ns *p* = 0.9257), *gpsB lcpC* (green circles, *n* = 103 cells, ns *p* = 0.7417), *gpsB ypfP* (pink circles, *n* = 114 cells, ^****^p < 0.0001), and *gpsB pbp4* (orange circles, *n* = 104 cells, ns *p* = 0.8137). The results were analyzed using a one-way ANOVA with multiple comparisons.

*S. aureus* encodes three LytR-CpsA-Psr (LCP) proteins within its genome: *lcpA, lcpB*, and *lcpC* (Over et al., 2011; Chan et al., 2013), all of which have a putative interaction with GpsB in a bacterial two-hybrid system (Kent, 2013). The LCP family of proteins catalyzes the transfer of WTA intermediates to the cell wall (Kawai et al., 2011; Chan et al., 2013). Severe phenotypic defects of *lcpA* mutants prevented the establishment of multiple mutant strains (Over et al., 2011; Chan et al., 2013), and no significant difference could be found in cell volume when comparing SH1000 *lcpB::ery* and SH1000 *lcpB::ery* Δ*divIVA gpsB::kan* (Supplementary Figure 3A). An *lcpC* mutation, which has previously been shown to have the smallest impact on cell growth and morphology (Over et al., 2011; Chan et al., 2013), was also tested. SH1000 *lcpC::ery* was found to be significantly smaller than wild-type SH1000, while SH1000 *lcpC::ery* Δ*divIVA gpsB::kan* was significantly larger than SH1000 (Figure 2A). This increase in cell size was observed in all stages of the cell cycle (Supplementary Figure 4A). Strains SH1000 Δ*divIVA lcpC::ery* and SH1000 *gpsB::kan lcpC::ery* were produced to deconvolve these results. Both *divIVA lcpC* and *gpsB lcpC* showed no significant differences in cell volume to SH1000 (Figures 2C, D). *lcpC* was smaller than the wild type, while *divIVA lcpC* and *gpsB lcpC* were the same size as SH1000. However, *divIVA gpsB lcpC* was bigger than the wild type. This suggests that a *divIVA* or *gpsB* mutation causes an increase in the *lcpC* mutant size (as *lcpC* alone is smaller than SH1000), and *divIVA gpsB* together have a cumulative effect upon the size increase of cells lacking *lcpC*. *divIVA gpsB lcpC* was complemented using a pKASBAR plasmid, containing native *gpsB*, which integrates into the *geh* locus of *S. aureus*. A control of an empty pKASBAR was also used. As expected, both *divIVA gpsB lcpC* and *divIVA gpsB lcpC geh::pKASBAR* were significantly larger than wild-type SH1000, whereas *divIVA gpsB lcpC geh::gpsB* was the same size (Supplementary Figure 5A).

TarO, the first enzyme in the biosynthetic pathway of wall teichoic acids (Soldo et al., 2002; Atilano et al., 2010), was shown to interact with both DivIVA and GpsB (Kent, 2013). SH1000 *tarO::ery* (Salamaga et al., 2021) and *divIVA gpsB tarO* both show a significantly increased cell volume compared to SH1000, and *divIVA* and *gpsB* mutations did not alter this phenotype (Supplementary Figure 3B).

Due to the apparent interaction between DivIVA and GpsB and proteins involved in WTA synthesis and display, a potential role for LTA, which has been shown to be involved in cell division (Gründling and Schneewind, 2007), was also investigated. As the synthesis of LTA is essential via the action of LtaS (Gründling and Schneewind, 2007), *ypfP*, which has an 87% reduction in LTA content (Fedtke et al., 2007), was investigated. SH1000 *ypfP::ery* has a significantly greater volume than SH1000, while *divIVA gpsB ypfP* was significantly smaller than wild type (Figure 2B), suggesting that LTA may be important in DivIVA or GpsB function. The decrease in cell volume was observed in all stages of the cell cycle (Supplementary Figure 4C). *divIVA ypfP* and *gpsB ypfP* were constructed to further analyse this phenotype. Both *divIVA ypfP* and *gpsB ypfP* show an increase in cell volume compared to SH1000 (Figures 2C, D), the same phenotype as *ypfP*. Therefore, the loss of *divIVA, gpsB*, and *ypfP* are all required for the reduced volume phenotype of the triple mutant. Complementation of *divIVA gpsB ypfP* with pKASBAR expressing native *gpsB* restored the increased cell volume phenotype (Supplementary Figure 5).

Both DivIVA and GpsB interact with PBP4 (Kent, 2013). SH1000 *pbp4::ery* had no significant difference in cell volume compared to parental SH1000, whereas *divIVA gpsB pbp4* has a significantly greater volume than SH1000 (Figure 2B). This increase in cell volume was only seen in cells with no or an incomplete septum (Supplementary Figure 4B). The results were deconvolved by constructing and analyzing *divIVA pbp4* and *gpsB pbp4*. *divIVA pbp4* shows a significantly greater increase in cell volume compared to SH1000 (Figure 2C). However, *gpsB pbp4* shows no significant difference in cell volume compared to the wild type (Figure 2D), demonstrating that only a loss of *divIVA* is required for the phenotype. This result was complemented using pKASBAR expressing native *divIVA* (Supplementary Figure 5B).

#### No functional links were found between *divIVA, gpsB*, and chromosome segregation

Previous research has shown a link between DivIVA and the segregation of chromosomes prior to cell division (Bottomley et al., 2017). The nucleoid occlusion protein Noc prevents the septa of a dividing cell to form over a chromosome, acting as an important checkpoint for chromosome segregation (Veiga et al., 2011). No chromosome segregation phenotype could be found for SH1000 Δ*divIVA gpsB::kan noc::ery*, based on DAPI staining, but a significant increase in cell volume was observed (Supplementary Figures 3C, 6).

DivIVA of *Corynebacterium glutamicum* binds to ParB and helps to orient the chromosome for cell division, as well as resulting in the mobilization of DivIVA (Giacomelli et al., 2022). While SH1000 *parB::ery* was slightly, but significantly, larger than SH1000, *divIVA gpsB parB* showed no significant volume differences to SH1000 or *divIVA gpsB* (Supplementary Figure 3D), and no abnormalities in chromosome segregation were observed (Supplementary Figure 6).

#### No relationship between *divIVA* with *ypsA* was observed

A previous study has shown that *gpsB* is in a syntenous relationship with *ypsA* within the genomes of Firmicutes, including *S. aureus* (Brzozowski et al., 2019), and conservation of such organization often indicates the shared function (Aravind, 2000; Huynen et al., 2000). Due to *gpsB* being encoded directly downstream from *ypsA*, we were unable to transduce both *ypsA::ery* and *gpsB::kan* into a single strain, so instead we analyzed *divIVA ypsA*. No differences could be found in cell volume (Supplementary Figure 3E) or chromosome segregation for *divIVA ypsA*.

### GpsB plays a role in *S. aureus* cell circularity

*S. aureus* has previously been shown to elongate during the cell cycle (Monteiro et al., 2015), resulting in a long axis and a short axis. Calculating the ratio between these two axes allows the extent of elongation to be calculated as previously reported (Reichmann et al., 2019) and acts as a measure of circularity. In this study, we calculated the ratio by dividing the short axis (axis perpendicular to the long axis) by the long axis (axis perpendicular to the septum). Using the short/long-axis ratio, a value of 1 indicates that the cell is perfectly circular, whereas the smaller the ratio, the more elongated the cell is.

**Figure 3:**
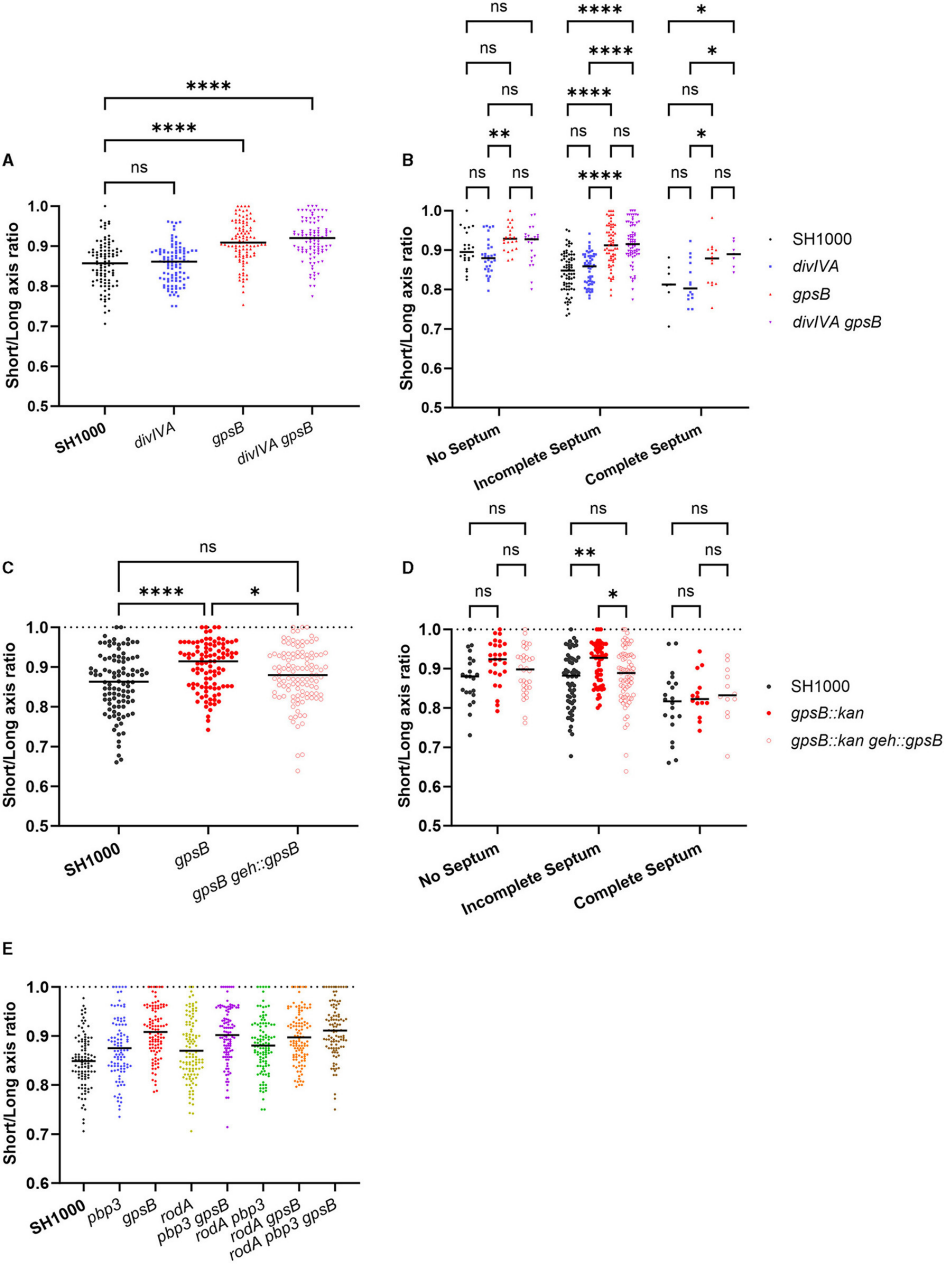
GpsB has a role in cell circularity. **(A)** The short-/long-axis ratio of SH1000 (black circles, *n* = 100 cells), *divIVA* (blue circles, *n* = 100 cells, *p* > 0.9999), *gpsB* (red circles, *n* = 101 cells, *p* < 0.0001), and *divIVA gpsB* (purple circles, *n* = 101 cells, *p* < 0.0001). Data were analyzed using a one-way ANOVA. **(B)** The short-/long-axis ratios from **(A)** organized by the stage of the cell cycle cells were in (*p*-values **p = 0.0024, ****p < 0.0001). Analyzed using a two-way ANOVA with multiple comparisons. **(C)** The short-/long-axis ratio of SH1000 (black circles, *n* = 100 cells), *gpsB* (red circles, *n* = 101 cells), and *gpsB geh::gpsB* (open red circles, *n* = 113 cells). Analyzed using a one-way ANOVA with multiple comparisons (ns *p* = 0.0903, *p = 0.0456, ****p < 0.00001). **(D)** The short-/long-axis ratios from **(C)** organized by the stage of the cell cycle cells were in (*p*-values *p = 0.0201, **p = 0.0024). Analyzed using a two-way ANOVA with multiple comparisons. **(E)** The short-/long-axis ratio of SH1000 (black circles, *n* = 100 cells), *pbp3* (blue circles, *n* = 99 cells), *gpsB* (red circles, *n* = 102 cells), *rodA* (yellow circles, *n* = 112 cells), *pbp3 gpsB* (purple circles, *n* = 107 cells), *pbp3 rodA* (green circles, *n* = 108 cells), *rodA gpsB* (orange circles, *n* = 111 cells), and *rodA pbp3 gpsB* (brown circles, *n* = 102 cells). The *p*-values for **(E)** are given in Supplementary Table 4.

The short/long-axis ratio was calculated for *divIVA* and *gpsB* mutants (Figure 3A). *divIVA* had no significant difference in short/long-axis ratio compared to SH1000, while both *gpsB* and *divIVA gpsB* had a significantly greater ratio, meaning that the cells are more circular, or less elongated, than wild-type cells. Both *gpsB* and SH1000 *divIVA gpsB* have significantly higher short-/long-axis ratios with incomplete septa compared to SH1000 and *divIVA*, but there is no difference with no septa (Figure 3B). Complementation with *gpsB* being expressed from the *geh* locus using pKASBAR (Bottomley et al., 2014) restored the elongation phenotype, with no difference in short/long-axis ratio between the wild-type and complemented strains (Figures 3C, D).

It has previously been reported that RodA and PBP3 form a cognate pair that is important for peripheral PG synthesis and the elongation of *S. aureus* (Reichmann et al., 2019). All single, double, and triple mutant permutations were created for *rodA, gpsB*, and *pbp3* and compared (Figure 3E). *pbp3* and *rodA pbp3* cells were significantly more circular than SH1000. *gpsB* was found to be significantly more circular than SH1000, *rodA, pbp3*, and *rodA pbp3*. *gpsB* showed no differences in circularity to *rodA pbp3 gpsB* (Figure 3E and Supplementary Table 4).

### GpsB influences PG synthesis and the localization of PBPs

**Figure 4:**
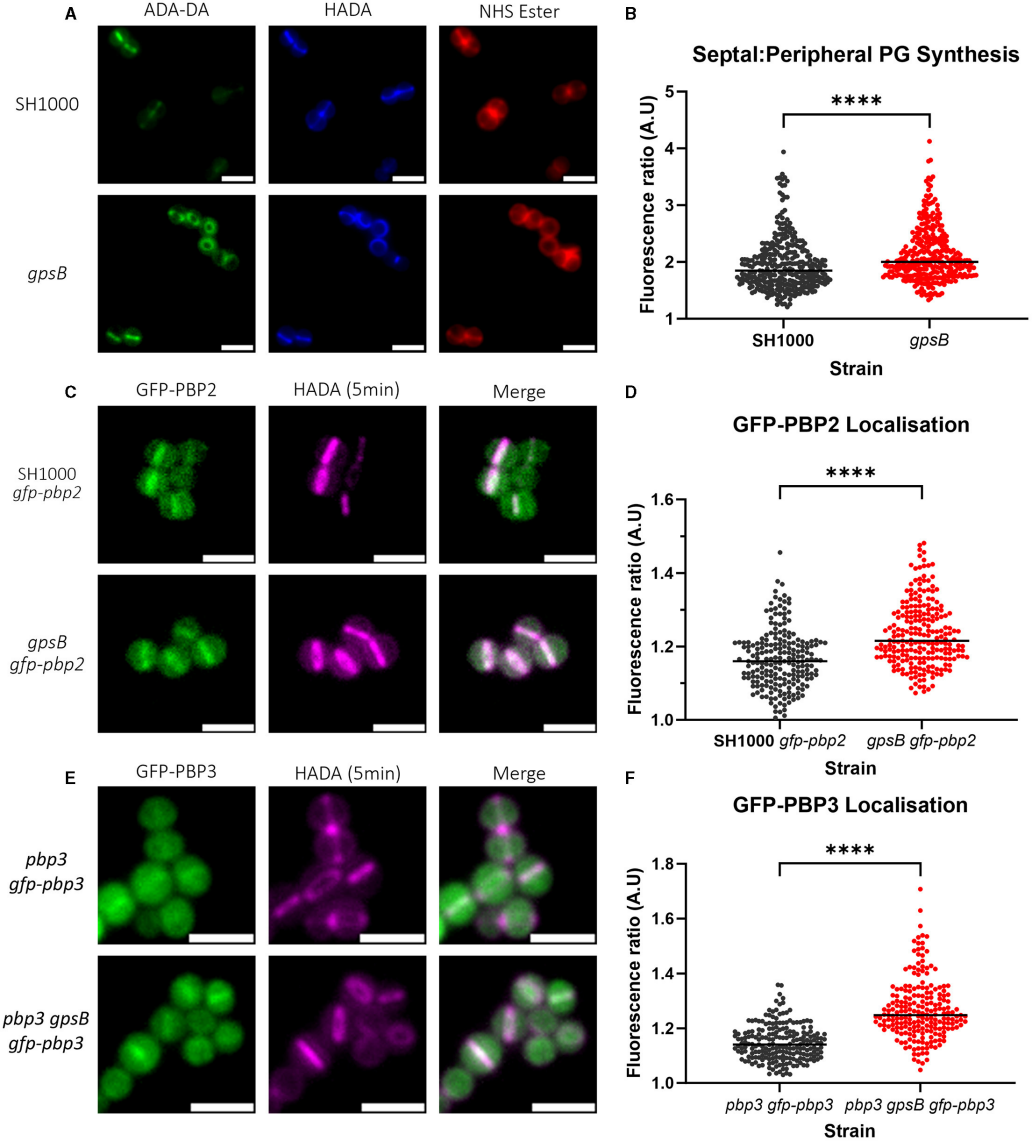
Role of GpsB in PG synthesis and PBP localization. **(A)** Representative micrographs showing ADA-DA (labeled with Atto 488, in green), HADA (in blue), and NHS Ester 555 (in red)-labeled cells of SH1000 and SH1000 *gpsB::kan* (scale bar represents 2 μm). **(B)** Fluorescence ratio (FR) values for the septal/peripheral ADA-DA signal in SH1000 (black circles, *n* = 322 cells) and *gpsB* (red circles, *n* = 312 cells). **(C)** Representative images of micrographs showing the localization of GFP-PBP2 in SH1000 *gfp-pbp2* and *gpsB gfp-pbp2* (scale bar represents 2 μm). **(D)** Fluorescence ratio values for the GFP-PBP2 signal in SH1000 *gfp-pbp2* (black circles, *n* = 209) and *gpsB gfp-pbp2* (red circles, *n* = 229). Representative images of micrographs showing the localization of GFP-PBP3 in **(E)** *pbp3 gfp-pbp3* and *pbp3 gpsB gfp-pbp3* (scale bar represents 2 μm). **(F)** Fluorescence ratio values for the GFP-PBP3 signal in *pbp3 gfp-pbp3* (black circles, *n* = 211) and *pbp3 gpsB gfp-pbp3* (red circles, *n* = 212). All were compared using the Mann–Whitney test (****p < 0.0001).

The localization of PG synthesis was determined by measuring the fluorescence ratio (FR) at the septum and the periphery of cells that have been sequentially labeled with ADA-DA and HADA (Figure 4A) for 5 min each to follow septal development (Tinajero-Trejo et al., 2022). The higher the FR, the greater the PG synthesis at the septum compared to the periphery. *gpsB* had a significantly higher FR than wild-type SH1000 (Figure 4B).

As PG synthesis is altered in *gpsB*, we determined PBP localization at the septum compared to the cell periphery using PBP fluorescent reporter fusions. PBP2 is the major PG transpeptidase in *S. aureus* responsible for the bulk of PG synthesis (Pinho et al., 2001a,b). Imaging SH1000 and *gpsB* expressing GFP-PBP2 (Figure 4C) showed a significant increase in FR for *gpsB* (Figure 4D), indicating that there is more GFP-PBP2 in the septum of dividing cells in the absence of GpsB.

PBP3 plays a role in the elongation of *S. aureus* due to off-septal PG synthesis (Reichmann et al., 2019). A GFP-PBP3 construct was made in pLOW under the control of the *Ppcn* promoter, which was then transduced into SH1000 *pbp3::spec* and SH1000 *pbp3::spec gpsB::kan*; therefore, the only expressed copy of PBP3 was GFP-PBP3. To test whether the fusion was functional, the circularity of SH1000 (Supplementary Figure 7A), *pbp3* (Supplementary Figure 7B), *pbp3 gfp-pbp3* (Supplementary Figure 7C), and *pbp3 gpsB gfp-pbp3* (Supplementary Figure 7D) was compared. *pbp3* is significantly more circular than SH1000. *pbp3 gfp-pbp3* was found to be significantly less circular than *pbp3* and was found to have no difference compared to SH1000 but could not complement the increased circularity of *gpsB* (Supplementary Figure 7E). *pbp3 gpsB gfp-pbp3* (Figure 4E) had a significantly higher FR for GFP-PBP3 than *pbp3 gfp-pbp3* (Figure 4F), demonstrating increased PBP3 at the septum in the absence of GpsB.

## Discussion

DivIVA and GpsB are paralogues in *S. aureus*, suggesting that they may have overlapping roles. The two proteins were found to not co-localize, and through screening, we noted interesting phenotypes for *divIVA pbp4, divIVA gpsB ypfP*, and *divIVA gpsB lcpC*. A change in morphology was also noted for *gpsB* mutants. *gpsB* was found to be more circular, or less elongated, than wild-type cells, a phenotype that was more pronounced than for a PBP3 mutant (Reichmann et al., 2019). This phenotype is due to an increased proportion of PG synthesis at the septum of *gpsB* cells, which was associated with an increased proportion of PBP2 and PBP3 localizing at the septum of *gpsB* cells. The results presented here suggest that GpsB plays a role in *S. aureus* PG synthesis regulation, specifically in regulating between septal and peripheral synthesis.

Both *pbp4* and *lcpC* combination knockouts were shown to produce larger cells (Figure 2). WTAs are responsible for the localization of PBP4 at the septum and for determining the level of PG crosslinking via the activity of PBP4 (Atilano et al., 2010). LcpC is involved in the ligation and display of WTA on PG after its synthesis. As DivIVA and GpsB both interact with TarO (Kent, 2013), an apparent link between DivIVA and GpsB with WTA is implicated. It has recently been demonstrated that GpsB directly interacts with TarG, is involved in the export of WTA, and was found to localize with the divisome complex (Hammond et al., 2022). GpsB, and currently also DivIVA, plays a role in linking WTA and cell division together, perhaps for the regulation of division. The increase in cell volume observed with these combination mutants could be due to the deregulation of PG synthases, potentially due to disconnect with WTA (Atilano et al., 2010). Despite GpsB not being a part of the *S. aureus* LTA synthesis machinery complex (Reichmann et al., 2014), in this study, we have suggested a link between DivIVA, GpsB, and LTA. LTAs play a role in septal placement as well as determining morphology, as cells lacking *ypfP* were misshapen (Kiriukhin et al., 2001; Gründling and Schneewind, 2007; Oku et al., 2009). A *ypfP* mutant was shown to be larger than SH1000, which is previously noted (Reichmann et al., 2014). However, cells that lacked *ypfP, divIVA*, and *gpsB* were significantly smaller, suggesting deregulation of cell growth, widening the molecules important for the function of both DivIVA and GpsB from WTA to teichoic acids in general. It has been suggested that GpsB acts as an adapter protein that brings together components of the divisome to regulate division (Cleverley et al., 2019). Our results suggesting a link between DivIVA, GpsB, WTA, and LTA further add evidence for this hypothesis.

Owing to the changes in volume being observed for the *pbp4, lcpC*, and *ypfP* combination mutants, it is possible that a change in PG synthesis is responsible. Interestingly, DivIVA has been shown to interact with PBP1 (Bottomley et al., 2017). With DivIVA having an interaction with DivIC (which itself also interacts with WTA), it is possible that they act in similar pathways. DivIC has been found to play a role in the septal formation and in the regulation and localization of PBP2 (Tinajero-Trejo et al., 2022). With DivIVA having a wide range of interactions, it is conceivable that DivIVA helps to regulate similar processes and brings together components of the divisome. The lack of a strong phenotype associated with the loss of *divIVA* implies that its function is redundant in the cell, or assisting the activity of other proteins, perhaps through stabilizing their interactions.

Previous studies have shown that, during the cell cycle, *S. aureus* elongates slightly (Monteiro et al., 2015), and this elongation is due, in part, to the activity of RodA and PBP3 incorporating PG to the side wall (Reichmann et al., 2019). While GpsB has been shown to only directly interact with PBP4 (Steele et al., 2011; Kent, 2013), it was found to be important for the localization of PBP2 and PBP3. As the localization of these PBPs is altered in a *gpsB* mutant, GpsB may occlude accumulation of these PBPs at the septum, allowing insertion of PG into the peripheral cell wall and subsequent elongation of the cell. Regulation of septal and peripheral PG synthesis has previously been described for *B. subtilis*, where direct interaction between PBP1 and MreC switched the cell between the two modalities of synthesis (Claessen et al., 2008; Tavares et al., 2008; Gamba et al., 2009). GpsB plays a similar role in rod-shaped *Listeria monocytogenes*, switching synthesis between the periphery and the septum by interacting with PBPA1 (Cleverley et al., 2016; Rismondo et al., 2017). In the ovococcoid *S. pneumoniae*, GpsB also regulates peripheral cell wall and septal synthesis. GpsB was shown to activate PBP2a and localize PBP2x to the late-stage septum, with a model suggesting that GpsB inhibited cell elongation by restricting the activity of PBP2b (Rued et al., 2017).

GpsB has been shown to interact with and stabilize FtsZ, concentrating its GTPase activity and helping to activate treadmilling for cytokinesis (Eswara et al., 2018). The transition between stages of cell division must be tightly controlled to prevent the chromosome from being damaged during segregation and ensure the two daughter cells are identical (Lund et al., 2018; Saraiva et al., 2020). As GpsB stabilizes Z-ring formation and then helps to activate treadmilling, as well as regulates PG synthesis at the septum and periphery, it could be possible that GpsB is regulating and bringing together components to temporally regulate cell division, ensuring that stages are occurring at the right time and order (Lenarcic et al., 2009). In *B. subtilis*, DivIVA is known to localize to negatively curved membranes via its N-terminal lipid-binding domain (Halbedel and Lewis, 2019). Such sites include the emerging division site due to the constriction of the membrane by FtsZ (Harry and Lewis, 2003; Eswaramoorthy et al., 2011), where it sandwiches the Z-ring by forming a ring on either side (Eswaramoorthy et al., 2011).

During consideration of our manuscript, another study on the role of GpsB has been published as a preprint (Costa et al., 2023). This study utilized *S. aureus* COL and found that cells lacking *gpsB* are rounder than the wild type. However, the authors show evidence that this is due to the mislocalisation of PBP2 and PBP4 more to the cellular periphery (Costa et al., 2023). Differences between studies are likely due to the strains used. Notably, COL is an MRSA, containing the non-native PBP2A, which is not present in the SH1000 strain (Horsburgh et al., 2002a).

Here, we have furthered the knowledge of *S. aureus* DivIVA and GpsB function, showing a link with teichoic acids. In addition, we have shown a role for GpsB in cell shape determination. We propose a model whereby GpsB plays a role in the elongation of cells by blocking the binding of PBPs (specifically 2 and 3) at the septum so that a greater proportion of PG synthesis occurs at the cell periphery, resulting in elongation.

## Data availability statement

The datasets presented in this study can be found in online repositories. The names of the repository/repositories and accession number(s) can be found at: ORDA database (10.15131/shef.data.23501532).

## Author contributions

JS, JH, and SF: designed research. JS, MC, MT-T, KW, BS, CP-R, and VL: performed research. JS, MT-T, KW, BS, JH, and SF: analyzed data. JS and SF wrote the manuscript. All authors contributed to the article and approved the submitted version.

## In memoriam

This study was dedicated to Mark Cooke, who sadly passed away before the project was completed.
