# Supplementary material for: The roles of GpsB and DivIVA in Staphylococcus aureus growth and division
Source: Front Microbiol. 2023 Aug 30;14:1241249. doi: 10.3389/fmicb.2023.1241249 (PMC10498921; doi:10.3389/fmicb.2023.1241249)
Supplement: Supplementary file 1 [file Data_Sheet_1.PDF]

Name	Genotype	Source
E. coli		
NEB5α		New England Biolabs
Staphylococcus aureus		
SH1000	Functional *rsbU*+ derivative of 8325–4	(Horsburgh et al., 2002)
SJF4925	SH1000 *gpsB::kan*	This Study
SJF4814	SH1000 Δ*divIVA*	This Study
SJF5093	SH1000 Δ*divIVA gpsB::kan*	This Study
SJF5640	RN4220 *gpsB::gpsB-mCherry kan*	This Study
SJF5643	SH1000 *gpsB::gpsB-mCherry kan*	This Study
SJF5299	SH1000 *ΔdivIVA geh::divIVA-gfp*	This Study
SJF5669	SH1000 *ΔdivIVA geh::divIVA-gfp gpsB::gpsB-mCherry kan*	This Study
SJF4899	SH1000 *ΔdivIVA geh::divIVA*	This Study
SJF4956	SH1000 *gpsB::kan geh::gpsB*	This Study
NE415	JE2 *lcpC::Tn(ery)*	(Fey et al., 2013)
SJF5448	SH1000 *lcpC::ery*	This Study
SJF5623	SH1000 *gpsB::kan lcpC::ery*	This Study
SJF5620	SH1000 Δ*divIVA lcpC::ery*	This Study
SJF5449	SH1000 Δ*divIVA gpsB::kan lcpC::ery*	This Study
SJF5743	SH1000 Δ*divIVA gpsB::kan lcpC::ery geh::pKASBAR*	This Study
SJF5744	SH1000 Δ*divIVA gpsB::kan lcpC::ery geh::gpsB*	This Study
SJF4425	SH1000 *pbp4::ery*	(Sutton et al., 2021)
SJF5624	SH1000 *gpsB::kan pbp4::ery*	This Study
SJF5621	SH1000 Δ*divIVA pbp4::ery*	This Study
SJF5492	SH1000 Δ*divIVA gpsB::kan pbp4::ery*	This Study
SJF5747	SH1000 Δ*divIVA pbp4::ery geh::pKASBAR*	This Study
SJF5748	SH1000 Δ*divIVA pbp4::ery geh::divIVA*	This Study
NE1663	JE2 *ypfP::Tn(ery)*	(Fey et al., 2013)
SJF5526	SH1000 *ypfP::ery*	This Study
SJF5625	SH1000 *gpsB::kan ypfP::ery*	This Study
SJF5622	SH1000 Δ*divIVA ypfP::ery*	This Study
SJF5527	SH1000 Δ*divIVA gpsB::kan ypfP::ery*	This Study
SJF5745	SH1000 Δ*divIVA gpsB::kan ypfP::ery geh::pKASBAR*	This Study
SJF5746	SH1000 Δ*divIVA gpsB::kan ypfP::ery geh::gpsB*	This Study
NE486	JE2 *noc::Tn(ery)*	(Fey et al., 2013)
SJF5442	SH1000 *noc::ery*	This Study
SJF5443	SH1000 Δ*divIVA gpsB::kan noc::ery*	This Study
NE1778	JE2 *lcpB::Tn(ery)*	(Fey et al., 2013)

SJF5446	SH1000 *lcpB::ery*	This Study
SJF5447	SH1000 Δ*divIVA gpsB::kan lcpB::ery*	This Study
NE1697	JE2 *ypsA::Tn(ery)*	(Fey et al., 2013)
SJF5469	SH1000 *ypsA::ery*	This Study
SJF5470	SH1000 Δ*divIVA gpsB::kan ypsA::ery*	This Study
NE772	JE2 *parB::Tn(ery)*	(Fey et al., 2013)
SJF5444	SH1000 *parB::ery*	This Study
SJF5445	SH1000 Δ*divIVA gpsB::kan parB::ery*	This Study
SJF5289	SH1000 *tarO::ery*	(Salamaga et al., 2021)
SJF5482	SH1000 Δ*divIVA gpsB::kan tarO::ery*	This Study
SJF4956	SH1000 *gpsB::kan geh::gpsB*	This Study
SJF4421	SH1000 *pbp3::ery*	(Wacnik et al., 2022)
SJF4422	SH1000 *pbp3::spec*	(Wacnik et al., 2022)
NE1598	JE2 *rodA::Tn(ery)*	(Fey et al., 2013)
SJF4837	SH1000 *rodA::spec*	This Study
SJF5596	SH1000 *pbp3::ery gpsB::kan*	This Study
SJF5579	SH1000 *rodA::spec pbp3::ery*	This Study
SJF5578	SH1000 *rodA::spec gpsB::kan*	This Study
SJF5597	SH1000 *rodA::spec pbp3::ery gpsB::kan*	This Study
SJF5659	SH1000 *pLOW-ppcn-gfp-pbp2*	(Tinajero-Trejo et al., 2022)
SJF5671	SH1000 *gpsB::kan pLOW-Ppcn-gfp-pbp2*	This Study
SJF5750	SH1000 *pbp3::spec pLOW-Ppcn-gfp-pbp3*	This Study
SJF5751	SH1000 *pbp3::spec gpsB::kan* pLOW-*Ppcn-gfp-pbp3*	This Study
RN4220	Restriction deficient transformation recipient	(Kreiswirth et al., 1983)
Ery, erythromycin resistance; Tet, tetracycline resistance; Kan, kanamycin resistance; Spec, Spectinomycin.		

Supplementary Table 1 Strains used in this study

Name	Relevant genotype/markers	Source
pKASBAR *ery*	pUC18 containing *attP* and an *ery* resistance cassette. Amp, Ery	(Bottomley et al., 2014)
pKASBAR *tet*	pUC18 containing *attP* and a *tet* resistance cassette. Amp, Tet	(Bottomley et al., 2014)
pKASBAR-*divIVA*	pKASBAR *tet* encoding the whole of *divIVA* with 178 bp upstream to include native promoter and ribosome binding site. Fragment synthesised and cloned into BamHI and EcoRI sites	This Study
pKASBAR-*gpsB*	pKASBAR *tet* encoding the whole of *gpsB* with 1170 bp upstream to include native promoter and ribosome binding site. Fragment synthesised and cloned into BamHI and BglII sites.	This Study
pKASBAR-*divIVA-gfp*	pKASBAR *tet* encoding 1000bp upstream of *divIVA,* followed by the whole of *divIVA*, *linker B* and *gfp*. Insert was synthesised by Genewiz, amplified using *pKB-divIVA-F/-R* primers, and cloned into pKASBAR *tet* cut with BamHI and EcoRI	This Study
pOB-*gpsB-mCherry*	pOB carrying *gpsB* with the stop codon removed and a *linkerA-mCherry* fusion attached followed by a kan^R cassette. Plasmid also has 658bp upstream of *gpsB* and 1000bp downstream to allow recombination. Ery, Amp, kan	GENEWIZ UK Ltd
piMAY	Temperature-sensitive Gram-positive replicon from pVE6007 with an *E. coli* replicon with tetracycline resistance cassette; Tet, Cm	(Monk et al., 2012)
piMAY *gpsB-ko*	piMAY carrying 1000bp upstream and 1000bp downstream *gpsB* with a kanamycin cassette between the two sequences. piMAY was cut with KpnI NotI. Tet, Cm, Kan	This Study
pMAD	*E. coli-S. aureus* shuttle vector with temperature-sensitive origin of replication in *S. aureus* and promoterless bgaB; Amp, Ery	(Arnaud et al., 2004)
pMAD *divIVA-ko*	pMAD carrying 1000bp upstream and 1000bp downstream of *divIVA.* pMAD was cut with BglII and EcoRI; Ery, Amp	This Study
pGL433	Vector carrying *kan* cassette suitable for selection in Gram-positive bacteria; Kan	(Wheeler et al., 2015)

pLOW	SK41-type low copy number plasmid; Amp, Ery	(Liew et al., 2011)
pLOW-*gfp-pbp2*	pLOW expressing a *gfp-pbp2* fusion under control of the penicillinase constitutive promoter (Ppcn); Ery	(Tinajero-Trejo et al., 2022)
pLOW-*gfp-pbp3*	pLOW expressing a *gfp-pbp3* fusion under control of the penicillinase constitutive promoter (Ppcn); Ery	Genewiz UK LTD This Study
Amp, ampicillin resistance; Ery, erythromycin resistance; Tet, tetracycline resistance; Kan, kanamycin resistance; Cm, chloramphenicol resistance.		

Supplementary Table 2 Plasmids used in this study

Oligonucleotide name (and restriction site)	Sequence (5' to 3')*	Use	Source
gpsB-up-F	atttctataaaagctacgtcactgtg	Amplifies the *gpsB* locus of *S. aureus*	This study
gpsB-down-R	tttcatacgtcgtatcaaggtc		This study
divIVA-up-F	tgcaacagttagttctttaaggttag	Amplifies the *divIVA* locus of *S. aureus*	This study
divIVA-down-R	aggcattaataacgtttctttgtaatc		This study
parB_F	cgaacccgtagacacctcat	Amplifies the *parB* locus of *S. aureus*	This study
parB_R	agcgattttagttgcaatgt		This study
ypfP_F	aaactaacggagggtggcta	Amplifies the *ypfP* locus of *S. aureus*	This study
ypfP_R	gcaatggatgtaactgttggc		This study
RodA_FWD	aaatctatagctgatatcactg	Amplifies the *rodA* locus of *S. aureus*	
RodA_REV	ttgactgtgattgtgaatc		
Noc_F	ggcaactcaagcgatgttca	Amplifies the *noc* locus of *S. aureus*	This study
Noc_R	acctttgaattgccagaaga		This study
lcpB_F	tttcatttgataattgcctcaca	Amplifies the *lcpB* locus of *S. aureus*	This study
lcpB_R	ggagtgcttcatagttttctcg		This study
lcpC_F	tagtaaaggagtggtgggat	Amplifies the *lcpC* locus of *S. aureus*	This study
lcpC_R	atcaccttctatttacgggc		This study
TnPbp3_F	tgatgaaaacattacagtgaatg	Amplifies the *pbp3* locus of *S. aureus*	(Wacnik et al., 2022)
TnPbp3_R	gtatcgccatatggatattttc		(Wacnik et al., 2022)
ypsA_F	acattgaacaactttctgcg	Amplifies the *ypsA* locus of *S. aureus*	This study
ypsA_R	actttgatcttcagaccact		This study
tarO_F	gcttcgaacatgtctgaatcgactc	Amplifies the *tarO* locus of *S. aureus*	(Salamaga et al., 2021)
tarO_R	gcagttacctttcgatataccaactg		(Salamaga et al., 2021)
pbp4-1	ctgcagaaaactttattttcaac	Amplifies a region of the *pbp4* locus of *S. aureus*	(Sutton et al., 2021)
pbp4-5	tatatagaactatcgatac taaac		(Sutton et al., 2021)
pMAD_*divIVA_1*	cgttacacattaactagacaatcatcaatcgctgcaattatc	Amplifies 1000bp upstream of *divIVA* for insertion into pMAD by Gibson assembly	This Study
pMAD_*divIVA_2*	tatttaattcttgttatcctccttaaatcattac		This Study
pMAD_*divIVA_3*	aggataacaagaattaaataaagacagacgc	Amplifies 1000bp downstream of *divIVA* for insertion into pMAD by Gibson assembly	This Study
pMAD_*divIVA_4*	gaattcgagctcccgggtactttttcgccatttacattg		This Study

pKASBAR_*divIVA_F*	cagctatgaccatgattacgaagtgaatcacactattgttg	Primers to amplify *divIVA* and incorporate into pKASBAR via Gibson assembly.	This Study
pKASBAR_*divIVA_R*	ctgccctttttttgccccggttacttcttagttgtttctgaatc		This Study
pKASBAR_*gpsB_F*	ggaaacagctatgaccatgattacgtatttagtaattatttaccaaatacagct	Primers to amplify *gpsB* and incorporate into pKASBAR via Gibson assembly.	This Study
pKASBAR_*gpsB_R*	cgggatccggccatgtaggccagataggcaagtacaagtatgtgttgt		This Study
piMAY_*gpsB_up_F*	agggaacaaaagctgggtactcaacaatagctttcttagttatcgcc	Primers to amplify 1000bp upstream of *gpsB* to incorporate into piMAY	This Study
piMAY_*gpsB_up_R*	tacgaggaattttactaaatacaaaagtttaactgtct		This Study
piMAY_*gpsB_down_F*	ggttcgctggtttttccacctcattagaaactttga	Primers to amplify 1000bp downstream of *gpsB* to incorporate into piMAY	This Study
piMAY_*gpsB_down_R*	gctccaccgcggtggcggccttaaatcaaattatatagagtgtt		This Study
pGL433_*kan_F*	gtatttagtaaaattcctcgtaggcgctcg	Primers to amplify the kanamycin resistance cassette from pGL433 to be incorporated into piMAY in between the *gpsB* upstream and downstream regions.	This Study
pGL433_*kan_R*	ggtggaaaaaccagcgaaccatttgaggtg		This Study
pKB-*divIVA-F*	ctgccctttttttgccccggatcatcaatcgctgcaattatc	Primers to amplify *divIVA-gfp* construct for incorporation into pKASBAR by Gibson assembly.	This Study
pKB-*divIVA-R*	cagctatgaccatgattacgttatttatacaattcgtccatacctaatg		This Study
*Restriction sites are in capitals and overhangs for the Gibson assembly in blue			

Supplementary table 3 Oligonucleotides used in this study

	SH1000	*pbp3*	*gpsB*	*rodA*	*pbp3 gpsB*	*rodA pbp3*	*rodA gpsB*	*rodA pbp3 gpsB*
SH1000		* 0.0284	**** <0.0001	ns 0.1328	**** <0.0001	** 0.0018	**** <0.0001	**** <0.0001
pbp3	* 0.0284		*** 0.0010	ns 0.9981	* 0.0164	ns 0.9971	ns 0.0970	*** 0.0002
gpsB	**** <0.0001	*** 0.0010		**** <0.0001	ns 0.9930	* 0.0102	ns 0.8371	ns >0.9999
rodA	ns 0.1328	ns 0.9981	**** <0.0001		*** 0.0009	ns 0.8649	** 0.0094	**** <0.0001
pbp3 gpsB	**** <0.0001	* 0.0164	ns 0.9930	*** 0.0009		ns 0.1061	ns 0.9983	ns 0.9453
rodA pbp3	** 0.0018	ns 0.9971	* 0.0102	ns 0.8649	ns 0.1061		ns 0.3864	** 0.0028
rodA gpsB	**** <0.0001	ns 0.0970	ns 0.8371	** 0.0094	ns 0.9983	ns 0.3864		ns 0.6249
rodA pbp3 gpsB	**** <0.0001	*** 0.0002	ns >0.9999	**** <0.0001	ns 0.9453	** 0.0028	ns 0.6249	

Supplementary Table 4 Statistics for Figure 3E

Derived *p* values for Figure 3E analysed using a one-way ANOVA with multiple comparisons.

Video 1 The localisations of GpsB and DivIVA

Videos showing Z-stacks of widefield fluorescence microscopy and maximum intensity projections for SH1000 *gpsB::gpsB-mCherry kanR ΔdivIVA geh::divIVA-megfp*. GpsB-mCherry (magenta), DivIVA-GFP (yellow) and HADA (cyan) are shown. **(A)** and **(B)** show different representative examples.

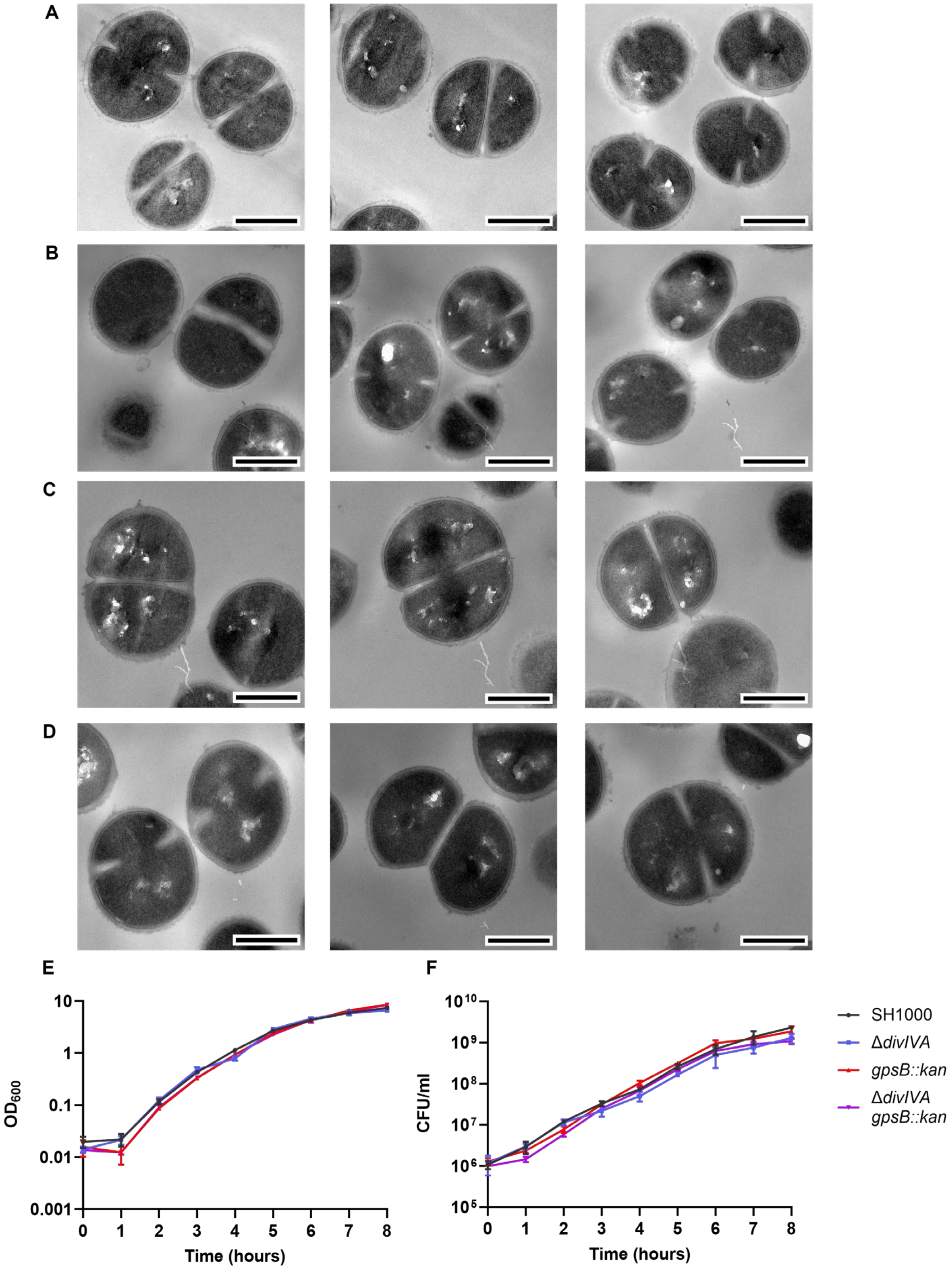

Supplementary Figure 1 Morphology and growth analysis of *gpsB and divIVA* mutants

Representative thin section TEM micrographs for **(A)** SH1000, **(B)** *divIVA,* **(C)** *gpsB* and **(D)** *divIVA gpsB* (scale bars represent 500 nm)*.* **(E)** Growth curves and **(F)** viability of SH1000 (black lines), *divIVA* (blue lines)*, gpsB* (red lines) and *divIVA gpsB* (purple lines). Bacterial cultures were prepared in triplicate and error bars show standard deviation.

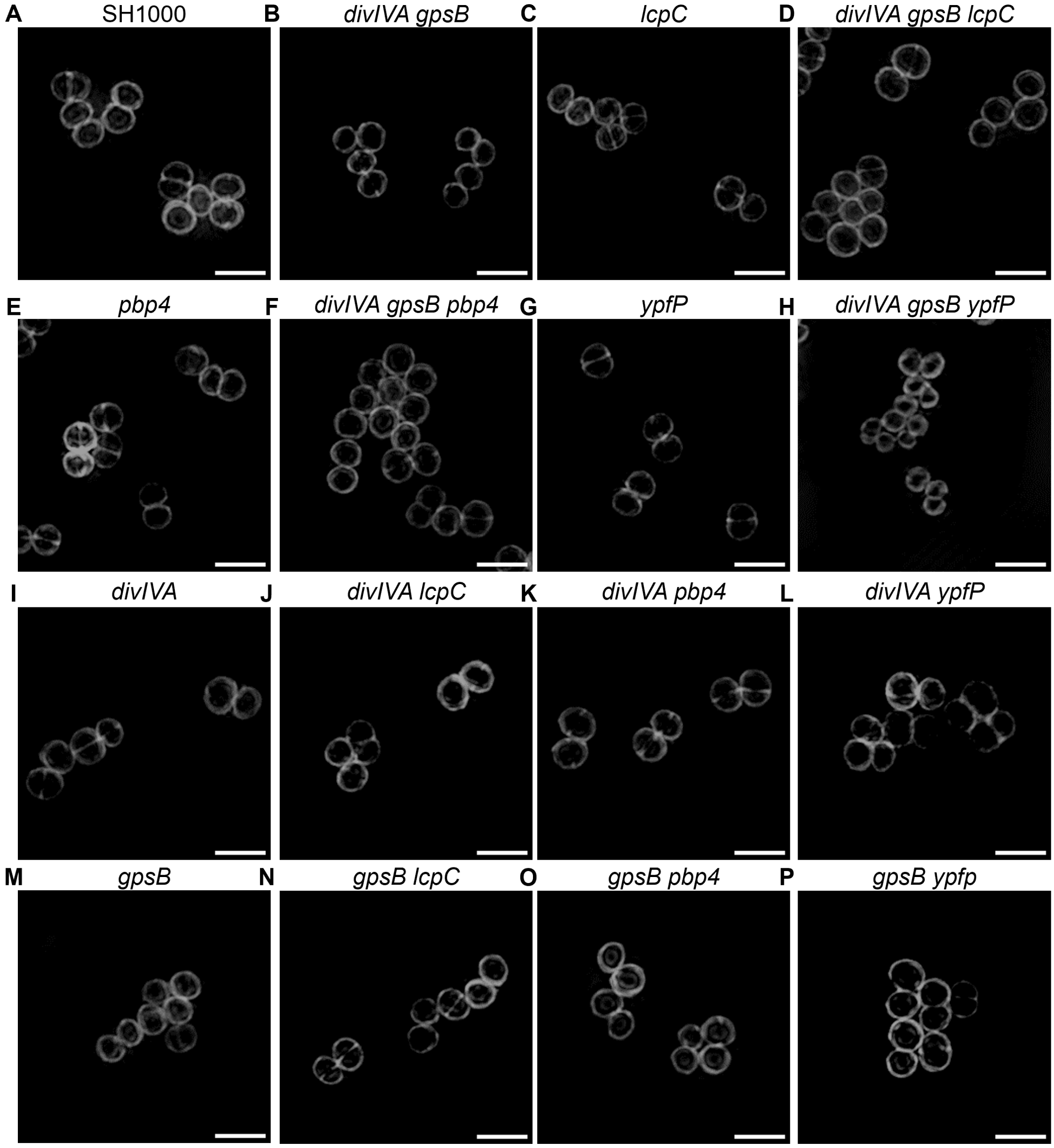

Supplementary Figure 2 Representative SIM micrographs of strains

Representative images of SIM micrographs of *S. aureus* strains **(A)** SH1000 **(B)** *divIVA gpsB* **(C)** *lcpC* **(D)** *divIVA gpsB lcpC* **(E)** *pbp4* **(F)** *divIVA gpsB pbp4* **(G)** *ypfP* **(H)** *divIVA gpsB ypfP* **(I)** *divIVA* **(J)** *divIVA lcpC* **(K)** *divIVA pbp4* **(L)** *divIVA ypfP* **(M)** *gpsB* **(N)** *gpsB lcpC* **(O)** *gpsB pbp4* **(P)** *gpsB ypfP* labelled with NHS Ester 555 (scale bars represent 2 µm).

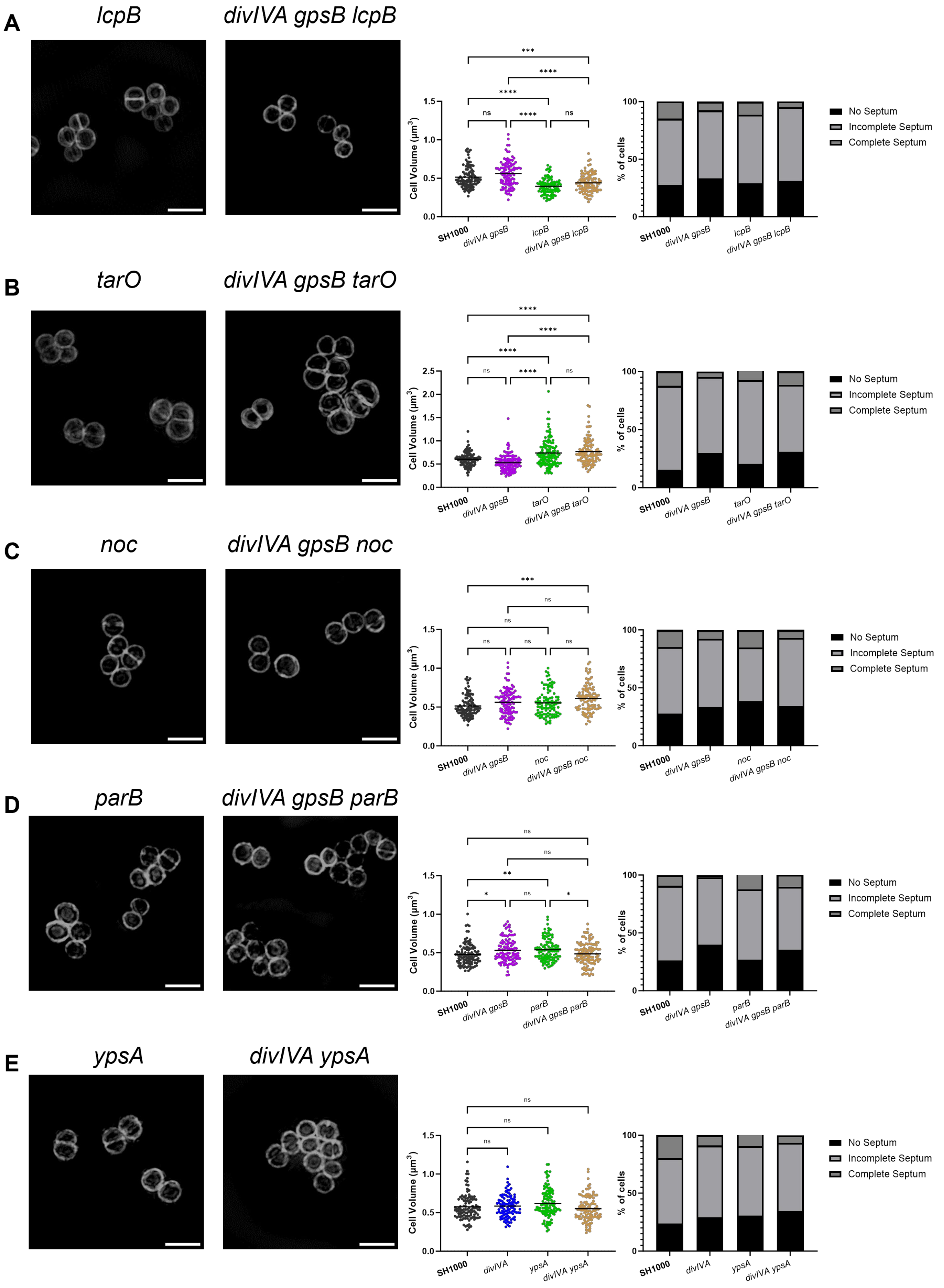

Supplementary Figure 3 Phenotypes of *divIVA gpsB* combination mutants

SIM images of mutants of genes of interest and triple mutants of *divIVA* and *gpsB* with the target genes, with cell volume and the percentage of cells in specific stages of the cell cycle recorded from these compared to SH1000 and *divIVA gpsB*. **(A)** *lcpB* and *divIVA gpsB lcpB.* **(B)** *tarO* and *divIVA gpsB tarO.* **(C)** *noc* and *noc divIVA gpsB.* **(D)** *parB* and *divIVA gpsB parB.* **(E)** *ypsA* and *divIVA ypsA.*

Results for cell volume analysis were analysed using a two-way ANOVA (* $p < 0.05$, ** $p < 0.005$, *** $p < 0.001$, **** $p < 0.0001$).

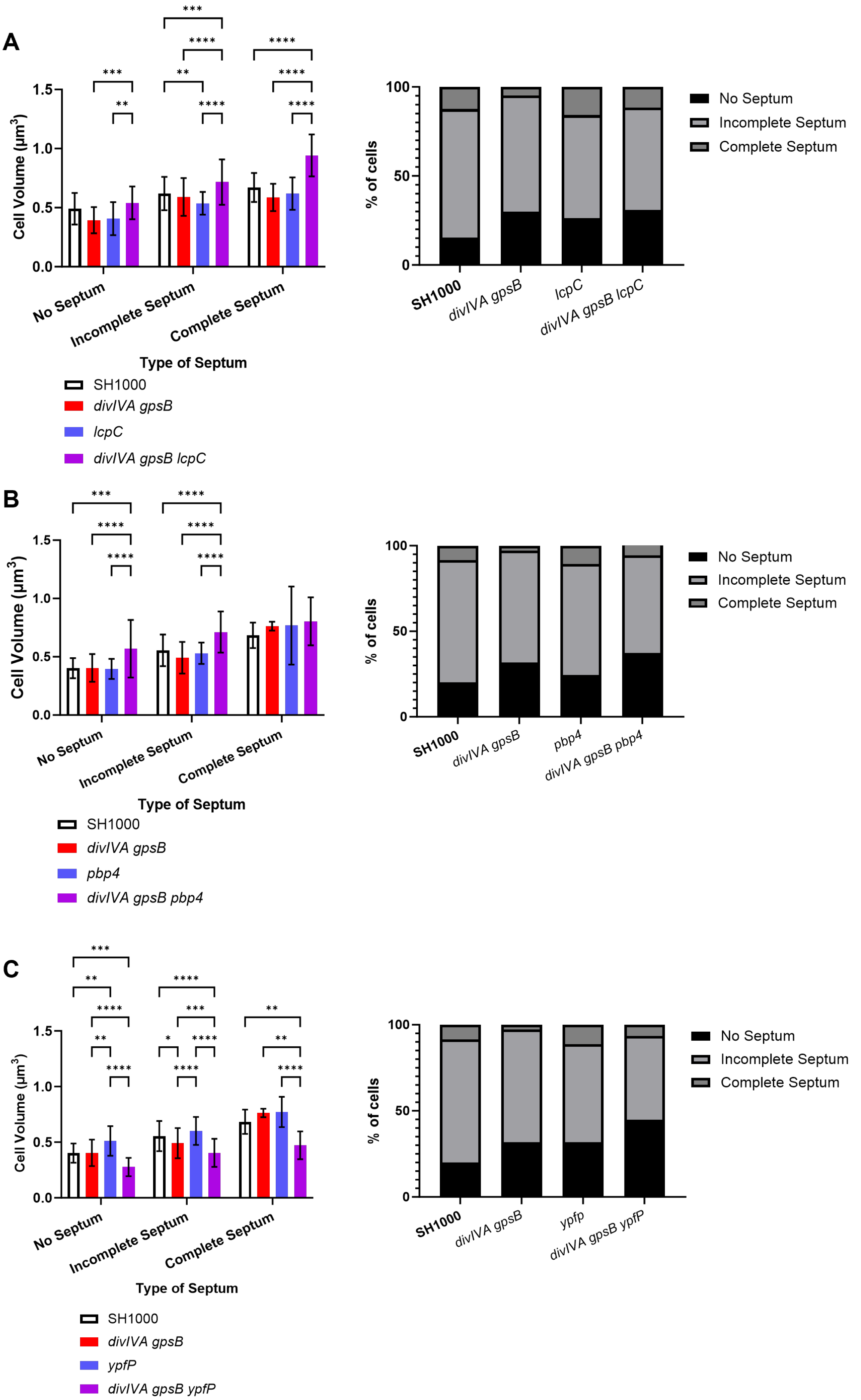

Supplementary Figure 4 Phenotypic analysis of *divIVA gpsB* combination mutants

Cell volumes per stage of the cell cycle and percentage of cells in specific parts of the cell cycle based on septum completion. **(A)** SH1000 (black bars, n = 104), *divIVA gpsB* (red bars, n = 124, *p* =

0.0028), *lcpC* (blue bars, n = 107), *divIVA gpsB lcpC* (purple bars, n = 104). **(B)** SH1000 (black bars, n = 105), *divIVA gpsB* (red bars, n = 107, *p* = 0.0028), *pbp4::ery* (blue bars, n = 102), *divIVA gpsB pbp4* (purple bars, n = 125). **(C)** SH1000 (black bars, n = 105), *divIVA gpsB* (red bars, n = 107), *ypfP* (blue bars, n = 113), *divIVA gpsB ypfP* (purple bars, n = 117). Results were analysed using a two-way ANOVA (* $p < 0.05$, ** $p < 0.005$, *** $p < 0.001$, **** $p < 0.0001$).

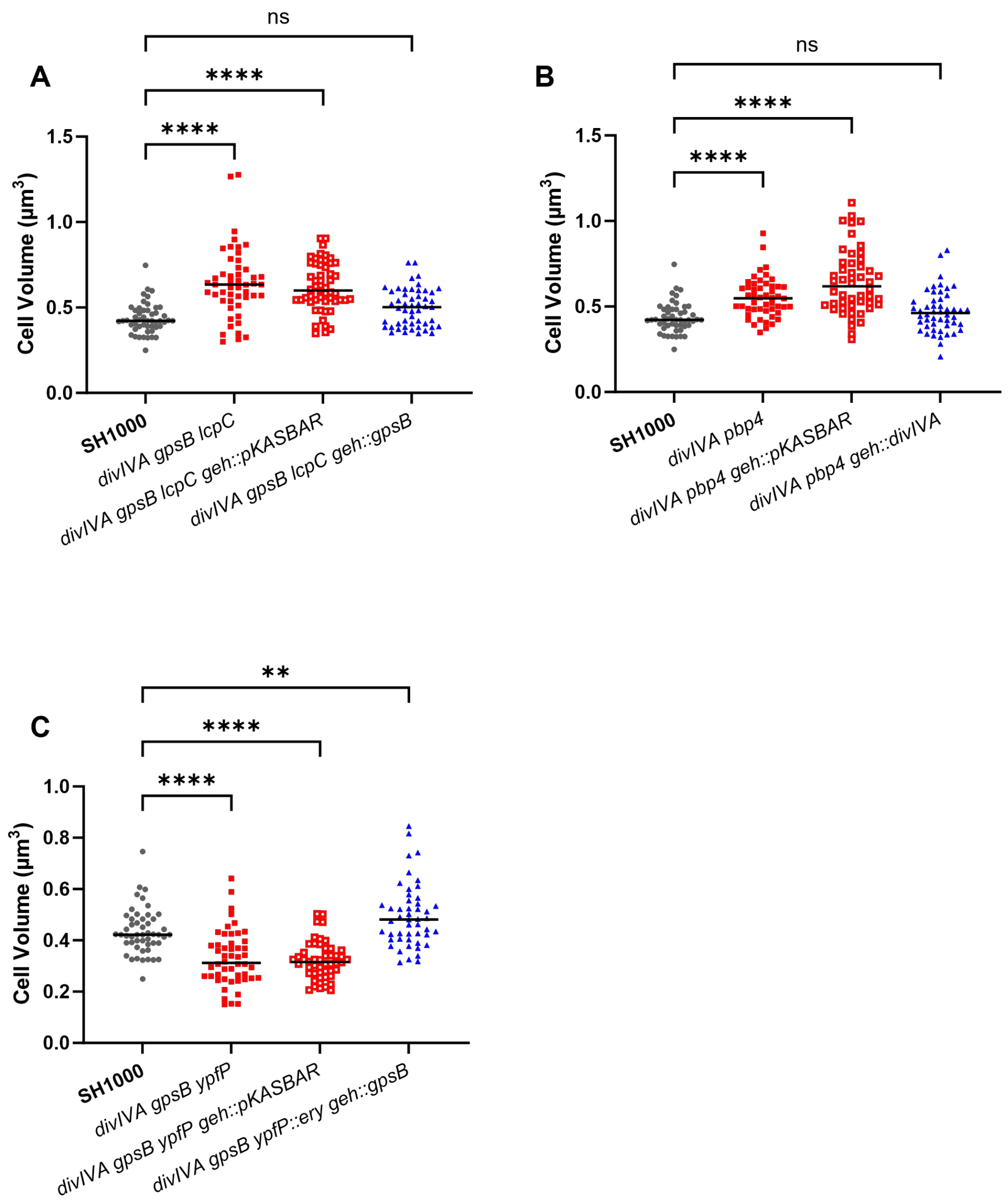

Supplementary Figure 5 Complementation of strains of interest

Complementation and cell volume measurements for strains of interest from Figure 2. **(A)** SH1000 (Black circles, n = 50), *divIVA gpsB lcpC* (red squares, n = 50, $p < 0.0001$), *divIVA gpsB lcpC geh::pKASBAR* (open red squares, n = 53, $p < 0.0001$) and *divIVA gpsB lcpC geh::gpsB* (blue triangles, n = 51, $p = 0.0638$). **(B)** SH1000 (Black circles, n = 50), *divIVA pbp4* (red squares, n = 50, $p < 0.0001$), *divIVA pbp4 geh::pKASBAR* (open red squares, n = 50, $p < 0.0001$) and *divIVA pbp4 geh::divIVA* (blue triangles, n = 50, $p = 0.4090$). **(C)** SH1000 (Black circles, n = 50), *divIVA gpsB ypfP* (red squares, n = 50, $p < 0.0001$), *divIVA gpsB ypfP geh::pKASBAR* (open red squares, n = 50, $p < 0.0001$) and *divIVA gpsB ypfP geh::gpsB* (blue triangles, n = 49, $p = 0.0095$).

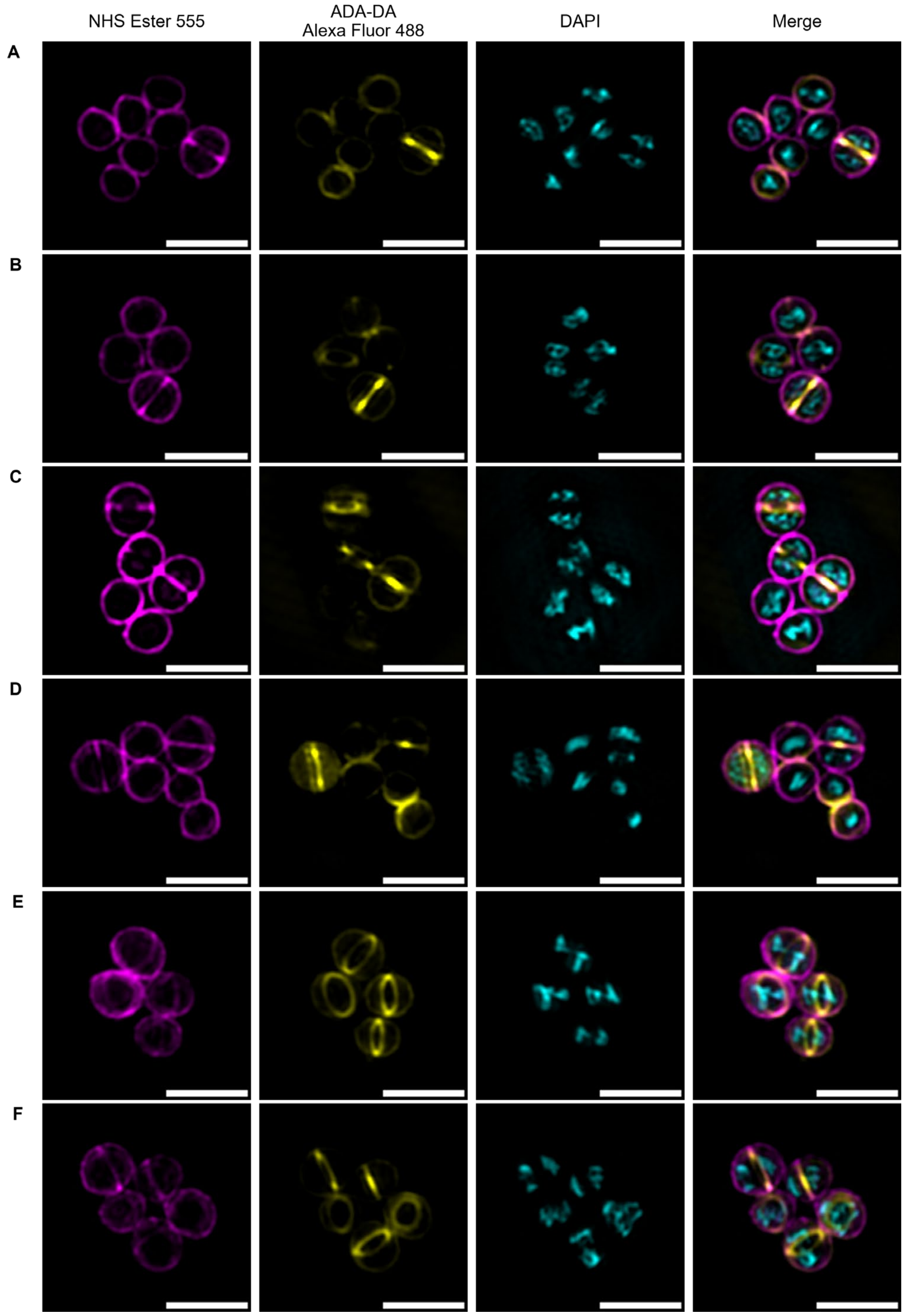

Supplementary Figure 6 Role of *divIVA* and *gpsB* in chromosome segregation

Representative images of SIM micrographs of *S. aureus* strains **(A)** SH1000 **(B)** *divIVA gpsB* **(C)** *noc* **(D)** *divIVA gpsB noc* **(E)** *parB* **(F)** *divIVA gpsB parB.* Cells are labelled with NHS Ester 555 (Magenta), ADA-DA (labelled with Atto488, yellow) and DAPI (Cyan) (scale bars represent 2 μm).

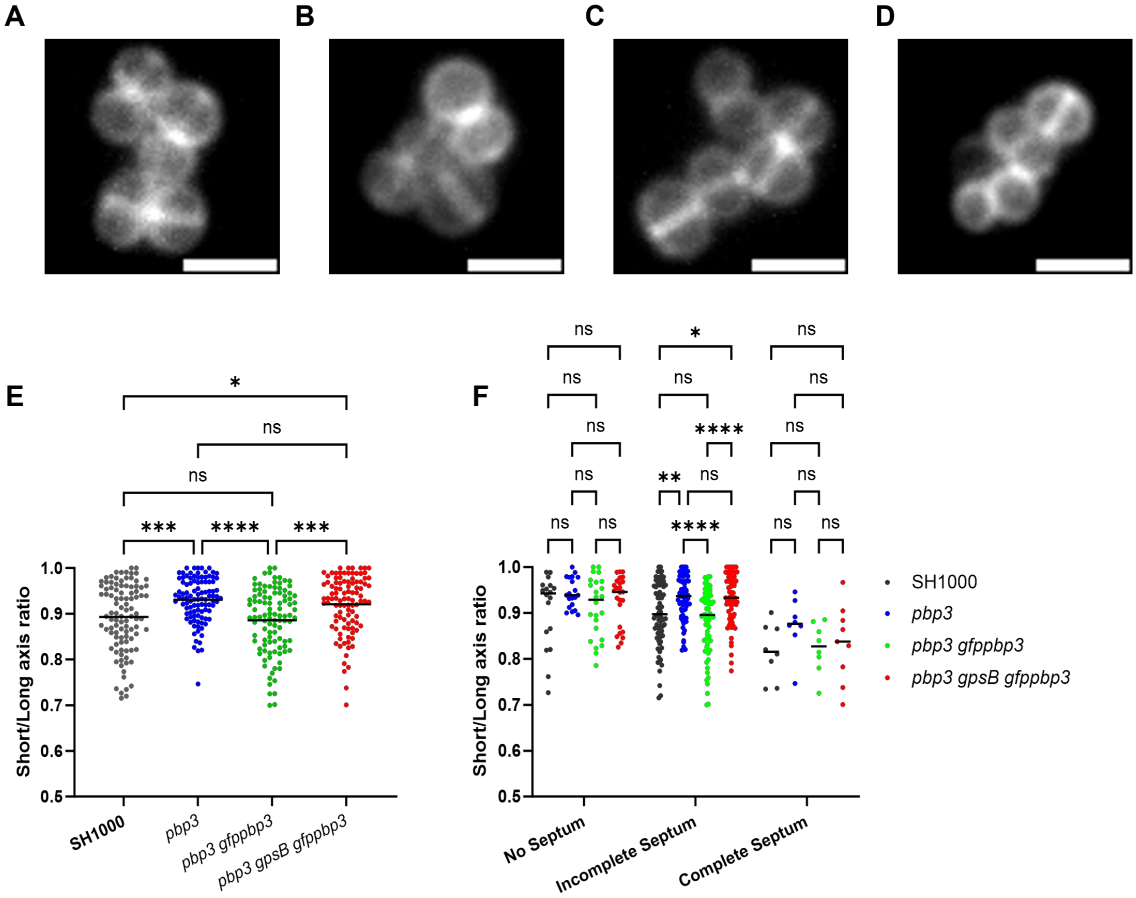

Supplementary Figure 7 Functionality of the GFP-PBP3 fusion protein

Micrographs for (A) SH1000 (B) *pbp3* (C) *pbp3 gfppbp3* (D) *pbp3 gpsB gfppbp3* each labelled with NHS Ester 555. Scale bars represent 2 μm. (E) The short/long axis ratio of SH1000 (black circles, n = 100), *pbp3* (blue circles, n = 101), *pbp3 gfppbp3* (green circles, n = 103) and *pbp3 gpsB gfppbp3* (red circles, n = 101). Data was analysed using a one-way ANOVA (* p = 0.0134, ** p = 0.0011, *** p = 0.0003 (SH1000 to *pbp3*) and 0.0007 (*pbp3 gfppbp3* to *pbp3 gpsB gfppbp3*), **** < 0.0001). **(F)** The short/long axis ratios from **(E)** organised by the stage of the cell cycle cells were in (p values * p = 0.0110, ** p = 0.0031, **** p <0.0001). Analysed using a two-way ANOVA with multiple comparisons.

References

Arnaud, M., Chastanet, A., and Débarbouillé, M. (2004). New vector for efficient allelic replacement in naturally nontransformable, low-GC-content, gram-positive bacteria. *Appl Environ Microbiol* 70, 6887–6891. doi: 10.1128/AEM.70.11.6887-6891.2004.

Boldock, E., Surewaard, B. G. J., Shamarina, D., Na, M., Fei, Y., Ali, A., et al. (2018). Human skin commensals augment Staphylococcus aureus pathogenesis. *Nat Microbiol* 3, 881–890. doi: 10.1038/s41564-018-0198-3.

Bottomley, A. L., Kabli, A. F., Hurd, A. F., Turner, R. D., Garcia-Lara, J., and Foster, S. J. (2014). Staphylococcus aureus DivIB is a peptidoglycan-binding protein that is required for a morphological checkpoint in cell division. *Molecular Microbiology* 94, 1041–1064. doi: 10.1111/mmi.12813.

Fey, P. D., Endres, J. L., Yajjala, V. K., Widhelm, T. J., Boissy, R. J., Bose, J. L., et al. (2013). A genetic resource for rapid and comprehensive phenotype screening of nonessential Staphylococcus aureus genes. *mBio* 4, e00537-00512. doi: 10.1128/mBio.00537-12.

Horsburgh, M. J., Aish, J. L., White, I. J., Shaw, L., Lithgow, J. K., and Foster, S. J. (2002). sigmaB modulates virulence determinant expression and stress resistance: characterization of a functional rsbU strain derived from Staphylococcus aureus 8325-4. *J Bacteriol* 184, 5457–5467. doi: 10.1128/JB.184.19.5457-5467.2002.

Kreiswirth, B. N., Löfdahl, S., Betley, M. J., O'Reilly, M., Schlievert, P. M., Bergdoll, M. S., et al. (1983). The toxic shock syndrome exotoxin structural gene is not detectably transmitted by a prophage. *Nature* 305, 709–712. doi: 10.1038/305709a0.

Liew, A. T. F., Theis, T., Jensen, S. O., Garcia-Lara, J., Foster, S. J., Firth, N., et al. (2011). A simple plasmid-based system that allows rapid generation of tightly controlled gene expression in Staphylococcus aureus. *Microbiology* 157, 666–676. doi: 10.1099/mic.0.045146-0.

Monk, I. R., Shah, I. M., Xu, M., Tan, M.-W., and Foster, T. J. (2012). Transforming the untransformable: application of direct transformation to manipulate genetically Staphylococcus aureus and Staphylococcus epidermidis. *mBio* 3, e00277-11. doi: 10.1128/mBio.00277-11.

Salamaga, B., Kong, L., Pasquina-Lemonche, L., Lafage, L., von Und Zur Muhlen, M., Gibson, J. F., et al. (2021). Demonstration of the role of cell wall homeostasis in Staphylococcus aureus growth and the action of bactericidal antibiotics. *Proc Natl Acad Sci U S A* 118, e2106022118. doi: 10.1073/pnas.2106022118.

Sutton, J. A. F., Carnell, O. T., Lafage, L., Gray, J., Biboy, J., Gibson, J. F., et al. (2021). Staphylococcus aureus cell wall structure and dynamics during host-pathogen interaction. *PLOS Pathogens* 17, e1009468. doi: 10.1371/journal.ppat.1009468.

Tinajero-Trejo, M., Carnell, O., Kabli, A. F., Pasquina-Lemonche, L., Lafage, L., Han, A., et al. (2022). The Staphylococcus aureus cell division protein, DivIC, interacts with the cell wall and controls its biosynthesis. *Commun Biol* 5, 1–13. doi: 10.1038/s42003-022-04161-7.

Wacnik, K., Rao, V. A., Chen, X., Lafage, L., Pazos, M., Booth, S., et al. (2022). Penicillin-Binding Protein 1 (PBP1) of Staphylococcus aureus Has Multiple Essential Functions in Cell Division. *mBio* 0, e00669-22. doi: 10.1128/mbio.00669-22.

Wheeler, R., Turner, R. D., Bailey, R. G., Salamaga, B., Mesnage, S., Mohamad, S. A. S., et al. (2015). Bacterial Cell Enlargement Requires Control of Cell Wall Stiffness Mediated by Peptidoglycan Hydrolases. *mBio* 6, e00660. doi: 10.1128/mBio.00660-15.
